# Photoactive layer based on T-shaped benzimidazole dyes used for solar cell: from photoelectric properties to molecular design

**DOI:** 10.1038/srep45688

**Published:** 2017-03-28

**Authors:** Beibei Xu, Yuanzuo Li, Peng Song, Fengcai Ma, Mengtao Sun

**Affiliations:** 1 College of Science, Northeast Forestry University, Harbin 150040, Heilongjiang, China; 2 Beijing Key Laboratory for Magneto-Photoelectrical Composite and Interface Science, School of Mathematics and Physics, University of Science and Technology Beijing, Beijing, 100083, China; 3 Department of Physics, Liaoning University, Shenyang, 110036, Liaoning, China; 4 Beijing National Laboratory for Condensed Matter Physics, Beijing Key Laboratory for Nanomaterials and Nanodevices, Institute of Physics, Chinese Academy of Sciences, Beijing, 100190, People’s Republic of China

## Abstract

Three benzimidazole-based organic dyes, possessing the same triphenylamine donors and cyanoacrylic acid acceptors with the bithiophene π-bridges combined in different nuclear positions of benzimidazole, were investigated in the utility of dye-sensitizer solar cells. The structure, molecular orbital and energy, absorption spectra and some important parameters (such as light harvesting efficiency (*LHE*), electron injection driving force, the electron injection time, chemical reactivity parameters, vertical dipole moment as well as interaction models of dye-I_2_) were obtained according to Newns–Anderson model and DFT calculation. The process and strength of charge transfer and separation were visualized with charge different density and index of spatial extent (S, D and Δq). Current work paid attention to the new T-shaped dyes to reveal the relation between the structure and photoelectric performance. Furthermore, nine dyes (substitution of alkyl chains and π-bridges) have been designed and characterized to screen promising sensitizer candidates with excellent photo-electronic properties.

With the rapid development of economy and the sharp rise in the global population, the demand for energy source is increasing. However, the traditional fossil energies (coal, oil and natural gas) exist several environmental problems. Solar energy has attracted immense attentions from both industrial and academic communities due to its inexhaustibility and no pollution^1^. In solar energy field, the dye sensitized solar cells (DSSCs) proposed by O’Regan and Grätzel^2^ have attracted ever-increasing attention because of their low cost and convenient synthesis^3^,^4^. The dye molecules play a key role in dye-sensitized solar cells: (a) they absorb sunlight to realize photoexcitation from the ground state to the excited state; (b) the electrons are injected into the conduction cluster, and holes are located on the molecule, accordingly achieve charge separation. Thus dyes serve as a vital function of energy conversion similar to chlorophyll in photosynthesis, and their performances directly affect the efficiency of the devices. Generally speaking, sensitizers can be divided into two categories, namely inorganic and metal-free organic dyes^5^,^6^. By the advantages of the wide absorption spectrum, the diversity of the molecular structure and low cost in comparison with rubidium, metal-free dyes are receiving more attentions.

Recently, benzimidazole^7^ and imidazole^8^,^9^ patterns have attracted extensive attentions as auxiliary donors and bulky hydrophobic moieties in solar cell, and some dyes have been demonstrated that they could improve light-harvesting properties and reduce charge recombination rates^7^,^8^,^9^. The dyes based on N-phenyl benzimidazole incorporated phenothiazine created bathochromic-shifted absorption and prevented undesirable charge recombination^10^, which were helpful for improvement of open-circuit photovoltage. Moreover, isomeric dyes^11^ differed in chromophores positions, which tuned spectrum response and affected their photovoltaic properties. Based on the above mention, three benzimidazole-based isomeric organic dyes(7a, 7b and 7c, possessing two triphenylamine donors and a cyanoacrylic acid acceptor) were synthesized by stoichiometrically controlled Stille or Suzuki−Miyaura coupling reaction^12^. The π bridge of dye 7a was bonded at C2 site, and dye 7b thiophene moiety was coupled to C4, and the π bridge of 7c was connected with C7 site. Among three dyes, T-shaped dye7a obtained the highest overall conversion efficiency (5.01%). In this article, we performed a detailed calculation to investigate optical absorption^13^ and charge transfer^14^,^15^ and recovery under surface and electrolytic. The relationship between structure and performance has been revealed. What’s more, the impacts of their butyl and substitution of π-bridges were also taken into consideration through designed dyes; moreover, uplifting results have been achieved including broadening absorption range, improving LHE, vertical dipole moment, etc.

We believe our calculated results can offer some guidance for the further research of T-shaped sensitizers possessing higher PCE (photo-to-electron conversion efficiency).

## Results

### Geometric Structures

**Figure 1:**
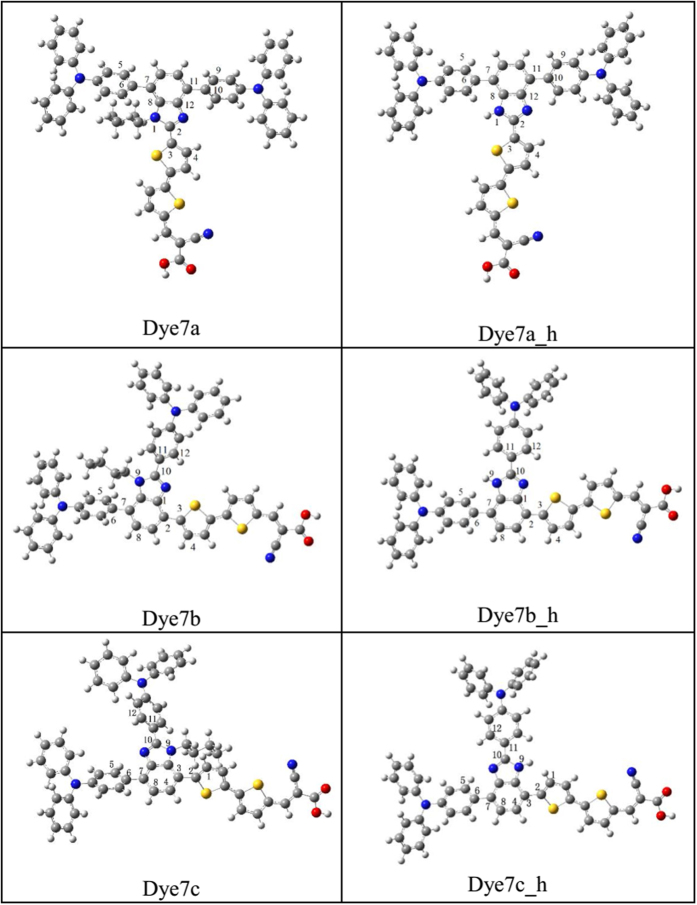
Names and chemical structures of all the sensitizers (where labels 2, 11, 7 represent C2, C4 and C7 for dye7a, respectively; labels 10, 2, 7 represent C2, C4 and C7 for dye7b, respectively; labels 10, 7, 3 represent C2, C4 and C7 for dye7c, respectively).

As shown in Fig. 1, dyes 7a, 7b and 7c have been successfully synthesized in the laboratory^12^, which possess the two triphenylamine groups donor, bithiophene π-bridge, a cyanoacrylic acid acceptor and butyl coupled with imidazole, respectively. The π bridge of dye 7a was coupled with C2 site, and two triphenylamine groups were bonded to C4 and C7 sites (see Fig. 1); thiophene moiety of dye 7b was combined with C4, and two triphenylamine were coupled in the C2 and C7 position, respectively; the π bridge of 7c located in C7, and two triphenylamine groups with C2 and C4 were connected. According to the geometric configuration of features, dye 7a belongs to T-cross-conjugated structure; as for 7b and 7c, triphenylamine moiety and the receptor are directly connected to the benzimidazole system through C4 and C7 sites, which viewed as the linear structures.

Table 1 showed that dihedral angles of three optimized dyes in both Gas and THF states. As shown, influence of the solution on the molecular geometry does not produce significant changes. To simulate a more realistic performance in dye-sensitized solar cells, the following calculation will be carried out in THF solution.

From the critical dihedral angles of dyes 7a → 7c, it was found that the dihedral angle formed between chromophore and π bridge(|C1-C2-C3-C4|) in 7a is computed to be 29.18°, and the dihedral angles of two triphenylamine groups and chromophores are 70.16° and 35.38°, respectively. For dye 7b, the dihedral angle formed between chromophore and π bridge(|C1-C2-C3-C4|) is 4.30°, and the dihedral angles of two triphenylamine groups and chromophores are calculated to be 61.04° and 41.77°, respectively. As for 7c, the dihedral angles formed between chromophore and π bridge(|C1-C2-C3-C4|) has increasing trend (the value of 65.43 °). As a result, the dihedral angles between benzimidazole and π bridge(|C1-C2-C3-C4|) decrease in the order of 7c > 7a > 7b. Noted that the typical T-shaped cross-conjugated structure (such as 7a) could be beneficial to effectively prevent π-π aggregation between the dye molecules, thus enhance their thermal stability^16^. The planar structure in 7b can strengthen the charge interaction from the donor and acceptor; however, the planarization of 7b will also affect the localization of the molecular orbital on the donor and may hinder the charge separation. As for the twisting structure of 7c, the severe distortion was originated from the butyl delinking the donor and acceptor, which may hinder the charge transfer and further weaken the short-circuit current.

### Frontier molecular orbitals

**Figure 2:**
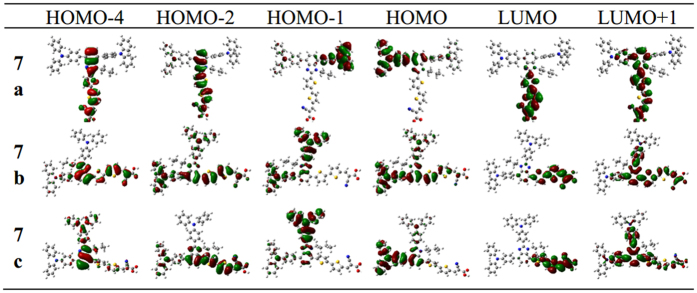
The frontier molecular orbitals calculated for 7a,7b and 7c.

The computed frontier molecular orbitals of three dyes are shown in Fig. 2. As shown in 7a, the HOMO-4, HOMO-2, LUMO and LUMO+1 are mainly located in the benzimidazole and acceptor structure, and the HOMO spread on the triphenylamine moiety attached at the C4 position, and HOMO-1 is distributed among mirror triphenylamine moiety at the C7. For 7b, almost all frontier orbitals have obvious nature of delocalization compared to 7a, i.e., HOMO and HOMO-2 of 7b are almost covering over the triphenylamine, bithiophene π-bridge and branch moieties, and LUMO and HOMO-4 are mainly located in the benzimidazole and acceptor segment. For 7c, LUMO+1 and HOMO-2 are delocalized over the whole molecule, and the LUMO has similar distribution with 7a; meanwhile the HOMO and HOMO-1 is located on triphenylamine moiety attached at the C4 and C2, respectively. From MOs distribution of 7a, 7b and 7c as described above, one can find that the performance of molecular located orbitals shows the trend 7a > 7c > 7b, which indicates the origination of electrons from the triphenylamine and chromophore (benzimidazole) via π bridge (bithiophene). The Best localization performance of dye 7a can make efficiently charge separation between donor and acceptor, and this distribution should be mainly due to its T-shaped structure which can isolate the donor and acceptor. However, 7b and 7c are inferior to 7a due to their linear structures that triphenylamine unit and acceptor are on one axis. Furthermore, for the linear structure of 7b and 7c, there is some difference for the localization performance. For example, the localization of 7c is better than 7b, which come from the butyl group on the side of acceptor significantly affecting the communication between donor and accepter by means of the harsh dihedral angle. If the butyl combines on the side of donor unit (7b), there is no interference for molecular framework, and the dihedral angle between them tends to planarization(4.30°), producing a greater range of delocalization.

**Figure 3:**
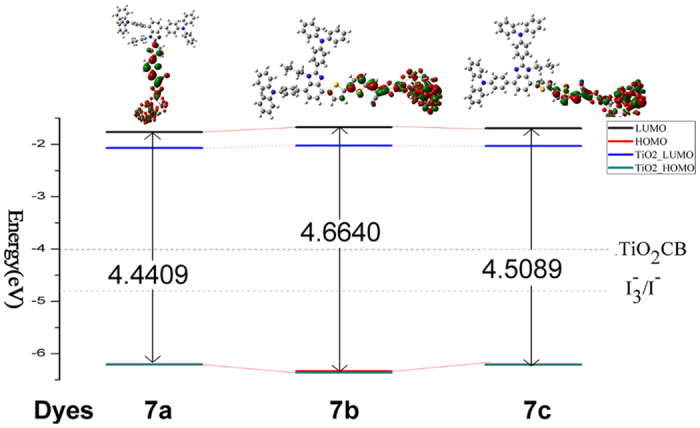
Schematic energy levels of sensitizers, together with the valence band of (TiO_2_)_9_ and I^−^/I_3_^−^ redox level.

To put deeper insight into the dependence of the electric properties on the geometry, the energy levels (*E*_gap_) of the frontier molecular orbitals for three dyes were calculated before and after adsorbing on the cluster, as shown in Fig. 3. For the isolated dyes in THF solvent, the values of *E*_gap_ are decreased in this order:7b(4.6640 eV) > 7c(4.5089 eV) > 7a(4.4409 eV), which is in line with the trend of orbital localization. Possibly, the electron density is strongly located between the donor and acceptor unit for HOMO and LUMO, respectively; the long ranged charge separation can easily take place upon excitation. In addition to orbital factors that affect energy gap of 7a, it is expected that the chromophore and π bridge binging two electronegative atoms N portion which facilitates electron transport^17^.

Figure 3 also shows the change of energy level of HOMO and LUMO before and after absorbing (TiO_2_)_9_. As shown, HOMO energy levels of 7a and 7a-TiO_2_ are −6.21 eV and −6.20 eV, therefore, there is little changed for two configuration 7a and 7a-TiO_2_; while for LUMO, 7a-TiO_2_ is −1.77 eV, which is larger than that of 7a (−2.07 eV). Similar trend is found for 7b and 7c (7b-TiO_2_ and 7c-TiO_2_). Some reports found that the HOMO level is closely related to the donor, and the LUMO level is mainly affected by the acceptor^18^; when the dyes adsorbed in the surface of titanium dioxide cluster, the LUMO and the TiO_2_ film will create relative strong electronic coupling due to the interaction between acceptor group and TiO_2_ surface, which make the LUMO of original molecules lower. Compared the energy level of three dyes, HOMO is in this order: dye7b(−1.67 eV) > dye7c(−1.70 eV) > dye7a(−1.77 eV), and the values of LUMOs follow a similar order: dye7b (−2.02 eV) > dye7c(−2.03 eV) > dye7a(−2.06 eV).

### Electronic absorption spectra

Before exploring nature of the absorption spectrum, we firstly carried out functional corrections with B3LYP^19^, CAM-B3LYP^20^ and ωB97xD^21^ functionals in THF solvent (see Table S1), which were compared with the experimental data. As shown in Table S1, the maximum absorption peak for 7a is 428 nm in the experiment, and the calculated absorption peaks are 519.01 nm with B3LYP, 451.31 nm with CAM-B3LYP and 439.55 nm with ωB97xD, respectively. The data obtained by ωB97xD is the closest to experimental one (11.55 nm), and CAM-B3LYP have a difference with the experimental data about 23 nm. And the calculated absorption peaks with B3LYP, CAM-B3LYP and ωB97xD functionals are 515.27 nm, 473.44 nm and 461.01 nm for dye 7b, and the data obtained by CAM-B3LYP is most accurate with respect to experiment (469 nm). Calculation with CAM-B3LYP on 7c also showed the smallest difference (5.97 nm) between experiment and theory. From the above discussion, it is clear that the data calculated by CAM-B3LYP have a better agreement with the experiment, and then simulated results in current work will be obtained from CAM-B3LYP in THF.

**Figure 4:**
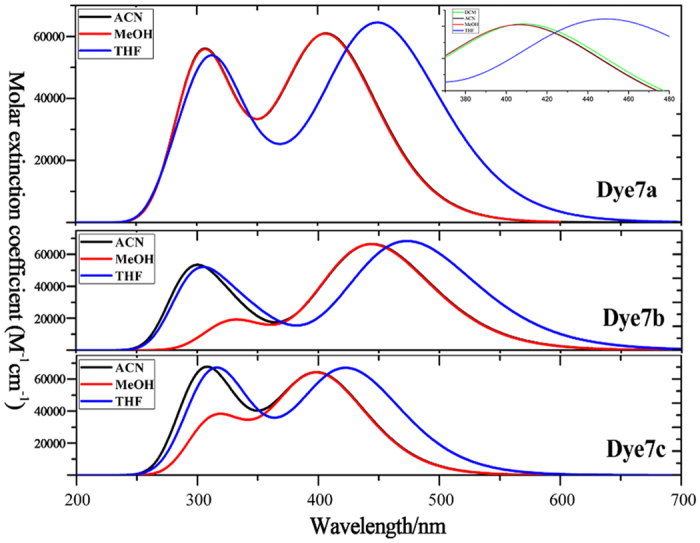
Absorption spectra of dye7a, dye7b and dye7c simulated in ACN (acetonitrile), MeOH (methanol), THF (tetrahydrofuran) and DCM (dichloromethane) solvents and specific spectral range from 370–480 nm for dye7a.

Figure 4 shows the absorption spectra of all dyes covering the visual and near-infrared regions, which are charactered as the two distinguish peaks. The one peak is at 300 nm, probably originated from the localized π-π* excitation of triphenylamine unit^22^; another absorption peak with the largest wavelength are predominantly attributed to the intramolecular charge transfer (ICT) from the triphenylamine donor to cyanoacrylic acid accepter. Because the positive proportional relation between oscillator strength and transition moment, the strongest absorption peaks correspond with the obvious electron transition moments (see Table S2). Furthermore, solvent effect has been discussed in THF, MeOH and ACN (see Fig. 4), in order to analyze red-shift performance and explicit the effect of polar solution on the absorption spectrum. And the polarity of the solvent can be quantified by the solvent parameter ∆*f*^23^, and the larger value of ∆*f* means the stronger polarity of the solvent. The polarity of the solvent follows the order of MeOH (Δ*f* = 0.393) > ACN(Δ*f* = 0.392) > THF(Δ*f* = 0.308), and the maximum absorption wavelength displays the same trend dye7b > dye7a > dye7c in all solvents. The analysis for a single molecule as 7a, the red-shifted in THF solution is most obvious; while spectra in ACN and MeOH solutions both show an obvious blue-shifted absorption, and the blue-shifted in MeOH solvent is slightly larger than that in the ACN solvent, which is related to the polarity of the solution in some degree. For more detailed studying the effect of polar solutions for 7a, we add another solvent (DCM) that the ∆*f* (0.320) is in the middle of the THF and the ACN, as shown inserted picture of Fig. 4. It is found that the absorption peak is in this order of THF > DCM > ACN, which supports the result that the polarity of the solvent have negative influence on absorption peak.

To understand the origins of absorption spectra, the electron transfer mechanism for the first six excited states were investigated, and each excited state was not a simple quantum state and could be described by a linear combination of several one-electron transition configurations. CI coefficients mean the primary HOMO–LUMO transition, which is responsible for an excited state. Here, the dominant configuration for each excited state and the excited state with f > 0.30 were discussed. Table 2 shows the excitation energies, oscillator strengths and CI expansion coefficient. For dye 7a, the first excited state corresponds to the electron transition of HOMO-2 → LUMO, which electrons move from benzimidazole to the bithiophene bridge (see the frontier molecular orbitals in Fig. 2). The state corresponding to a typical intramolecular charge transfer (ICT) model, and its maximum absorption peak is 451.31 nm^3^ (*f* = 1.5569). For S2 state, the electron transition is from HOMO to LUMO, displaying electron transfer from triphenylamine unit coupled at C4 position to π bridge, also assigned as ICT excitation, and its maximum absorption peak is 371.15 nm (*f* = 0.3023). For a higher excited state, the S4 state from HOMO to LUMO+1 reflects electron passing from triphenylamine unit bonding at C4 to benzimidazole chromophore. As for S5 state, it is mainly contributed to electron transition from HOMO-4 to LUMO, and this state has a similar electron transition pattern as S1. Thus we concluded that the maximum absorption of 7a is made up of the S1, which have the electronic transition from HOMO-2 to LUMO. The other peak at 300 nm is mainly made up of the S4 and S5, which corresponds to the electron transition of HOMO → LUMO+1 and HOMO-4 → LUMO.

The similar linear structures of 7b and 7c, the important excited state were analyzed and compared. For dye 7b, the first excited state is originated from HOMO to LUMO, namely electron transfer from triphenylamine units to the direction of cyanoacrylic acid, termed as ICT excitation with the maximum absorption peak of 473.44 nm (*f* = 1.6875). The S2 state composed of electron transition from HOMO-1 to LUMO is also a typical the ICT model from donor to the accepter, and its maximum absorption peak is 347.03 nm (*f* = 0.3747). Also S5 state possesses same transition mode as S2. It was worth noting that CI configuration of S1 state for 7c differs from that of 7b, namely, electron transition are composed of HOMO-2 → LUMO; for HOMO-2 the location of electron density on chromophore and acceptor certainly will result in the change of electron density in molecular framework. The electron transition of HOMO → LUMO+1 corresponding to S4, which is ICT state, and maximum absorption peak is 322.92 nm (*f* = 0.7094). For S5 state, however, the electron density change between HOMO-1 and LUMO+1 can be termed as a mixture of π-π* and ICT due to the delocalization of LUMO+1 over the entire molecule.

### Charge transfer properties

**Figure 5:**
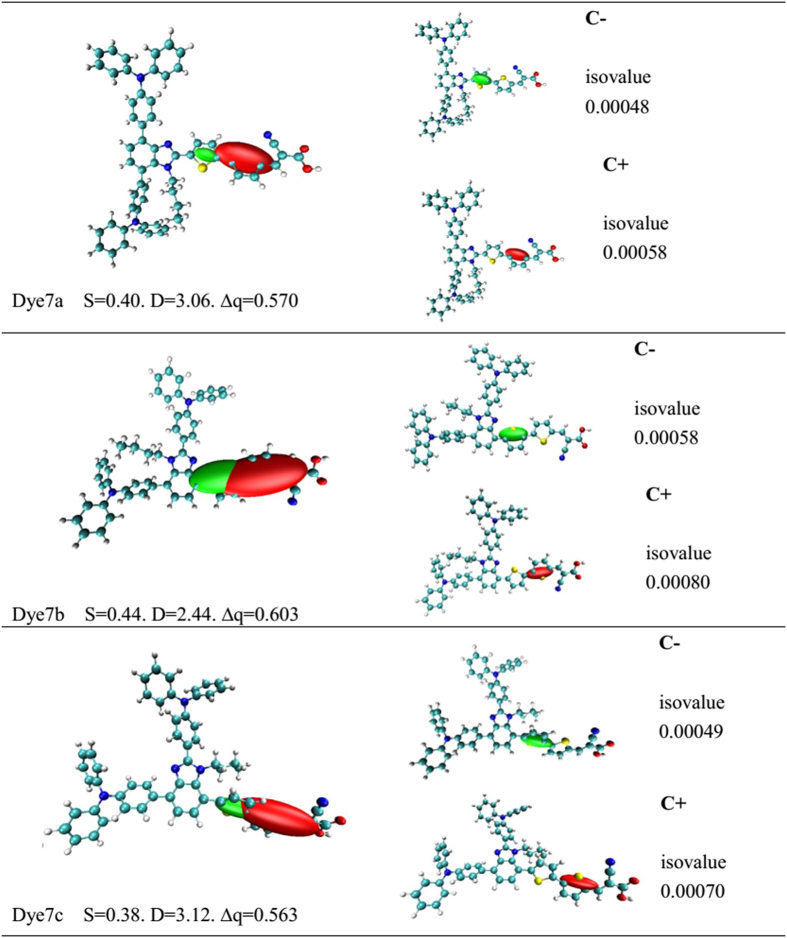
Graphical ellipsoid representations of CT which associated centroids of hole and electron (C+/C−)^a^; (^a^Computed difference in total density from S0 to S1 for all the dyes (isovalue 0.000493 a.u.), performed in THF solvent using CAM-B3LYP functional together with 6–31 G(d) basis set. D is the electron transfer distance (Å), S is overlaps between the regions of density depletion and increment, ∆q is the fraction of electron exchange (|e-|)).

For the dyes possessing push-pull structure, the significant charge separation is one of the common characteristics that guarantee a high overall efficiency of photo-to-electron conversion^24^,^25^. The first state of three dyes was all ICT states, and charge density difference^26^ shows the change of electron density upon excitation (see Supporting materials in Figure S1). As shown in Figure S1, there is no clear distinction of charge separation for 7a and 7c. In order to quantitatively describe the strength of ICT, index of spatial extent method provides the three additional parameters^27^,^28^ (as shown Fig. 5) including S (overlap integral between C+ and C−), D(the distance of electron transfer [Å]) and Δq (the fraction of electron exchange (|e-|)). The D is defined as an distance length between positive centroid and negative centroid, 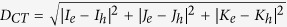, where I, J and K stand for three dimensional directions. *I*_*e*_ and *I*_*h*_ stand for The charge density along a certain orientation, for instance, 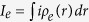 for electron and 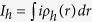 for hole, respectively. Overlap integral S expresses the overlap distance between hole and electron (C+ and C−): 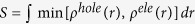, where *ρ*^*hole*^ (*r*) and *ρ*^*ele*^ (*r*) are the density distributions of hole and electron, respectively.

As shown in Fig. 5, calculated overlap distance is calculated to be 0.40 for 7a, 0.44 for 7b and 0.38 for 7c; the tendency is 7b(0.44) > 7a(0.40) > 7c(0.38). Among them, the value of S in 7c is the smallest owing to the twisted charge transfer between triphenylamine units and bithiophene bridge. The value of S in 7a is smaller than that of dye 7b due to the advantage of the T type inverted-hook structure. The strong charge separation make the value of S decreased. As mentioned in the frontier orbits section, it seems that the main occupied molecular orbitals and unoccupied molecular orbitals of 7a and 7c display well location upon donor and acceptor, and the density change of molecular orbitals upon excitation support the result of overlap integral S. For three dyes, the larger value D is, the distance between centroid of hole and electron is longer, and dye with larger value D exhibits a good charge separation. Calculated value of D is in this order: 7b(D = 2.44 Å) < 7a(D = 3.06 Å) < 7c(D = 3.12 Å), and the results have opposite sequence compared with S. Upon the analysis of amount of transferred charge (Δq), the tendency is 7b(Δq = 0.603) > 7a(Δq = 0.570) > 7c(Δq = 0.563). Noted that there is a larger amount of electron exchange between the ground state and the first excited state for 7b and 7a; but for 7c, introducing bithiophene unit at the C7 site of benzimidazole reduces the amount of electron exchange. From the maximum absorption wavelength in the section of Electronic absorption spectra, the order of red-shift is consistent with the squeeze of Δq.

In summary, the calculated results confirmed that the distance of electron transfer can reflect the strength of overlap integral from C+ to C− in degree. For the distance of charge separation, 7c is slightly better than 7a probably due to severe twisting structure, as supported by overlap value of S. Some reports indicated that the advantages of D and S may reduce charge recombination on organic dyes besides excellent charge separation^29^. At the same time, the associated ellipsoids (C+ and C−) can be easily visualized upon an electronic excitation, as depicted in Fig. 5 (the green and red colors represent the centroid of hole (C+) and the centroid of electron (C−) during S0 → S1). Centroid of hole regions can be seen as the structure of the offering electron (donor), and centroid of electron regions can be seen as the main electron-rich structure (acceptor). Figure 5 shows the distribution of C+/C− of three dyes (isovalue0.000493 a.u.) and individual analysis of C+/C− for each dye. As shown, the positive and negative centroid for T-type 7a are located on the two thiophene of bridge, and centroid of hole is located on the first thiophene, meaning that this thiophene take partly donor role and the second one as acceptor; the linear structural 7b and 7c have similar tendency.

### Factors influencing *J*_*SC*_

Short circuit current density (*J*_*SC*_) is an important factor affecting the overall conversion efficiency (η) upon photoexcitation, which can be estimated from the three parameters: light harvesting efficiency (*LHE*) of dye, the injection efficiency of the electrons in the excited state and the regeneration efficiency of the dye. Here, the charge collection efficiency has little difference because of the same semiconductor electrode (usually TiO_2_)^30^. The harvesting efficiency (*LHE*) can be calculated according to equation 6, and comparable assessment for the injection efficiency of the electrons in the excited state and the regeneration efficiency of the dye can be obtained from calculation of electron injection (Δ*G*^*inject*^) and the driving force for dye reorganization (∆*G*^^reg^^)^31^. The related data is listed in Table 3, and the *LHE* values are 0.9723 (7a), 0.9795(7b) and 0.9768 (7c), and the three values are so approximate that this parameter has little effect on *J*_*SC*_.

Based on Equation (7) and (9), the Δ*G*^*inject*^ and ∆*G*^^*reg*^^ can be estimated. The ∆*G*^^*reg*^^values of 7a, 7b and 7c are far more than 0.20 eV (2.2009 eV, 2.3626 eV and 2.2213 eV), indicating that the quantity is sufficient for dye reorganization according to ref. 32. As for Δ*G*^*inject*^, the injection efficiency of the electrons in the excited state (Φ_*inj*_) tends to 1 when the Δ*G*^*inject*^ is greater than 0.20 eV^33^. Thus, it can be considered that the three dyes have the same injection efficiency only judging from the Δ*G*^*inject*^. To further evaluate the performance of the electron injection for dye/TiO_2_ compounds, the electron injection lifetime (*τ*_inj_) was calculated by the Newns–Anderson model for adsorbates on the surface, which is served as the criterion to judge whether electron injection is energetically efficient or not. The *τ*_inj_ is inversely proportional to the electron injection efficiency Φ_*inj*_ simulation, obtained from the energy shift of the adsorbate’s LUMO after the dye was adsorbed on the semiconductor, which is simulated by the following equations^34^,^35^:


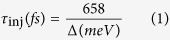



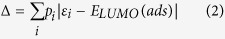


where Δ is energetic broadening, *p*_*i*_ is the adsorbate portion of every molecular orbital, *ε*_*i*_ is orbital energy and *E*_*LUMO*_ (*ads*) (eV) is the energy of the adsorbate’s LUMO. This method could accurately simulate the electron transfer process for the dye/TiO2 interface (the related data listed in Table 3). As shown, the results clearly confirm that an ultrafast and effective electron injection process occurs for each dye, because all the *τ*_inj_ values are in femtoseconds (fs). Dye 7a obtained the fastest injection lifetime of 3.96 fs, indicating the advantage in electron transfer process for 7a, which reached a good agreement with the experimental *J*_*SC*_.

**Figure 6:**
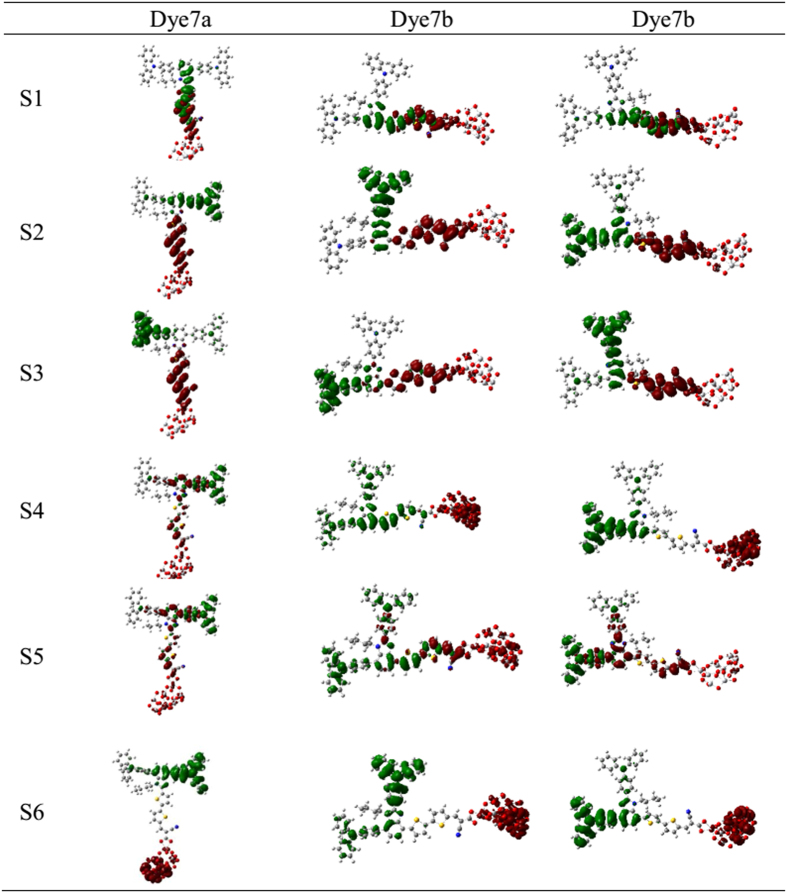
Electron density difference plots of electronic transition for all the dyes bonding with TiO_2_.

The charge density difference (CDD) were used to study the path of electron transfer for dyes-TiO_2_, as shown Fig. 6 (the green represents the hole density, and red stands for the electron density). For the S1 state, the hole density in 7a, 7b and 7c locates mainly on the benzimidazole, and electron density locates mainly on the cyanoacrylic acid segment, which have mixture in thiophene units. This result means the electron is transferred from the benzimidazole chromophore via bithiophene to cyanoacrylic acid (ICT mode), and the first thiophene in bridge serve as donor, which is in accordance with the index of spatial extent method. For the S2 and S3 state, the distribution of hole density is found in triphenylamine moiety and electron density is in the thiophene units, and thus the bithiophene actually plays a role of acceptor. Evidently, it is a significant ICT excitation. As for the S4 and S6, three dyes have several difference, i.e., 7a and 7c have significant tunneling effect, where the hole density concentrates on triphenylamine units, and electron density is located in TiO_2_ cluster, thus the charge directly translates from the triphenylamine unit to the cluster surface. However, the hole density for the S4 of 7b is almost located on the whole molecule, and electron is injected into the cluster. For S5 state of three dyes, common character is found that holes and electrons are delocalized throughout the molecular structure, and the charge is transferred from the triphenylamine via thiophene groups to the cyanoacrylic acid.

Table 3 lists the reorganization energy of three dyes, and the hole and electron reorganization energy based on Equation (11) and (12) can affect the *J*_*SC*_, that is, the smaller *λ*_total_ value is obtained, charge-carrier transport rates will be the faster^36^. Calculated hole reorganization energy (*λ*_h_) for 7a and 7c are significantly better than 7b, and the electron recombination energy (*λ*_e_) of 7b (0.44 eV) is slightly smaller than 7a and 7c (0.48 eV). The lifetime (τ) of the first excited state (S1) is also an important factor that affects the efficiency of charge transfer. A dye with a longer lifetime in the excited state is expected to be more facile for the charge transfer^37^. The excited state lifetimes of the dye sensitizers can be calculated via the equation: *τ* = 1.499/(*fE*^2^), where *E* (cm^−1^) is the excitation energy of the different electronic states and *f* is oscillator strength of the electronic state^38^. The calculated lifetime (τ) of the first excited state are listed in Table 3, they followed the order of 7a(2.64 ns) > 7b(2.57 ns) > 7c(2.26 ns). The results indicate that dye 7a remains stable in the cationic state for a longer time, which engenders a larger charge transfer efficiency and then enhances short circuit current density probably. As a result, the approximate *LHE* and sufficient ∆*G*^^reg^^and Δ*G*^*inject*^ cause little difference for 7a,7b and 7c. Hence, the electron injection time (*τ*_inj_) and the lifetime (τ) of the first excited state played a leading role in short current density of the three dyes.

### Factors influencing *V*_oc_

**Figure 7:**
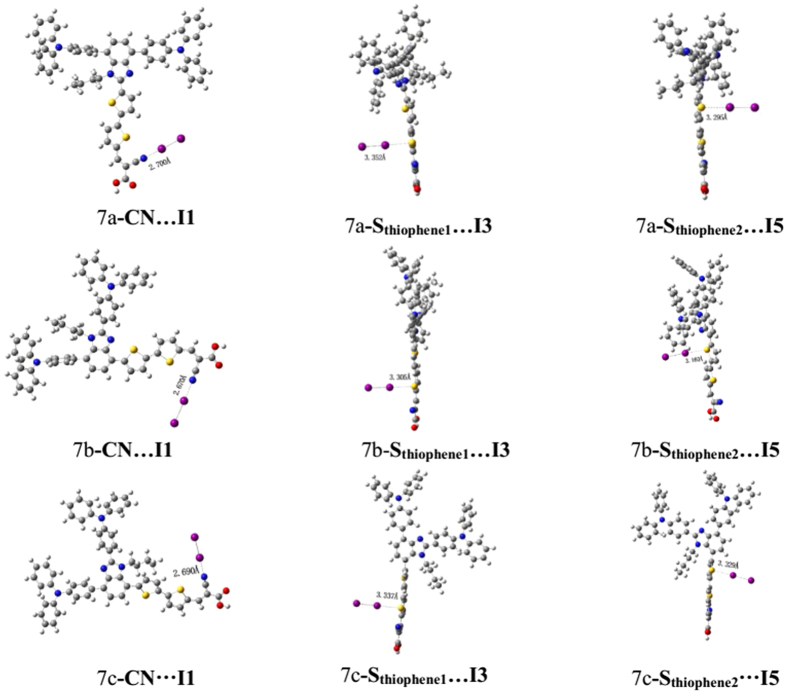
Geometries of the optimized dyes-I_2_ complex.

The open-circuit photovoltage (*V*_oc_) is also critical to the overall light conversion efficiency, and *V*_oc_ can be approximately estimated by the following equation^30^:


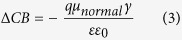


where *ε*_*0*_and *ε* are the permittivity of the vacuum and the dielectric constant of the organic monolayer, respectively; *q* is the electron charge. The *μ*_normal_ is the vertical dipole moment of the compound molecule in the surface of the semiconductor (where the surface of TiO_2_ is parallel to the yz plane, and x axis direction corresponding to the *μ*_normal_ (see Supporting materials in Figure S2)). Table 4 shows that the *μ*_normal_ for 7a, 7b and 7c is 12.5154D, 11.1531D and 2.1166D, respectively. The larger *μ*_normal_ could cause a larger value of *V*_oc_; therefore, 7a is expected to realize the highest photovoltage. Dye7b possesses comparatively better *μ*_normal_ than 7c, and 7b is expected to create a larger photovoltage; but the relatively higher *V*_oc_(0.71 V) was obtained for 7c, which may be originated form the distorted dihedral angle between π-bridge and chromophore that somehow blocks electron reorganization. Certainly, the only vertical dipole moment is not comprehensive to measure the open circuit voltage. There are two process of electron recombination^39^: (a) the injected electron in the conduction band of TiO_2_ could recombine with the redox couple (I^−^/I_3_^−^) in the electrolyte, which is the main route of electron photovoltage losses; (b) the injected electron in TiO_2_ recovers into oxidized organic dyes. Since dye molecules tend to adsorb on the TiO_2_ surface, I_2_ prefers to bind hetero atoms (O, N, S etc.) into the dye molecules forming halogen bond through a non-covalent interaction^40^, and the process will reduce the electron recovery from TiO_2_ into I^−^/I_3_^−^. Accordingly, we used I_2_ represent I^−^/I_3_^−^ to measure the electron recombination on the TiO_2_ surface. We considered the indirect interaction (the dye^…^I_2_ model) and construct three structures to scan active position. The optimized dye^…^I_2_ structures of 7a, 7b and 7c are shown in Fig. 7. Three interactions were shown: a) the interaction models (I_2_ binds to N of the CN moiety) are labeled I1-I2; b) bonding to atom S of the thiophene unit neighboring the accepter, named by I3-I4; c) connecting to the S (S_thiophene2_) of the thiophene moiety near benzimidazole, labeled as I5-I6. The distances of I-X (X = I, N and S) are listed in Table S3. For all dye^…^I_2_ complexes, the calculated results indicate the intramolecular I-I bond lengths are close to the covalent radii (2.86 Å), and the distances of I-X (X = N and S) are larger than the covalent radii (2.86 Å), while smaller than the net Van der Waals radii (3.53 Å).

The length of CN^*…*^I1 shows this order: 7a(2.700 Å) > 7c(2.690 Å) > 7b(2.670 Å), which is well agreement with the sequence of S_thiphene1^*…*^_I3, i.e., 7a(3.352 Å) > 7c(3.337 Å) > 7b(3.305 Å)). Therefore, active atoms (N and S) in the acceptor and conjugated bridge obey the same trend. However, for the S (S_thiophene2_) of the thiophene moiety near donor, there is little different contrast trend, i.e., 7c(3.329 Å) > dye7a(3.295 Å) > dye7b(3.160 Å). In term of the separation distance of iodines, the bond length orders of I1-I2 is 2.877 Å for 7a, 2.882 Å for 7c and 2.879 Å for7b, respectively, that is to say, 7a < 7c < 7b, and this trend is opposite to the interaction of dye^…^I_2_. So the increasing interaction between dye and iodines will result in increasing separation distance of iodines.

By analysis of interaction between dye and iodines, it was found that for all interaction model, the interaction lengths of 7b are obviously smaller than those of 7a and 7c, meaning that the fast electron recombination will appear in electrolyte. 7a can make the I_2_ away from the surface of titanium dioxide so that inhibit the local concentration on the semiconductor surface, as a consequence reduced charge recombination. This charge recombination can be supported by NBO method^41^. Table 4 shows that amount of charge migration for system of I2 followed the tendency (except I1-I2): 7b > 7c > 7a, and the relatively large charge migration will lead to the more loss of electron. For 7b, electrons are more likely to migrate towards the electrolyte, and the reason is that excessive planarization can interact with electrolyte easily and hardly keep off electrolyte from semiconductor, thereby reduce the circuit voltage. Finally, it was found that the calculated *μ*_normal_ and dye^…^I_2_ interaction are mainly agreement with the experimental *V*_oc_ (7a(0.73 V) > 7c(0.71 V) > 7b(0.70 V)).

### Chemical reactivity parameters

**Figure 8:**
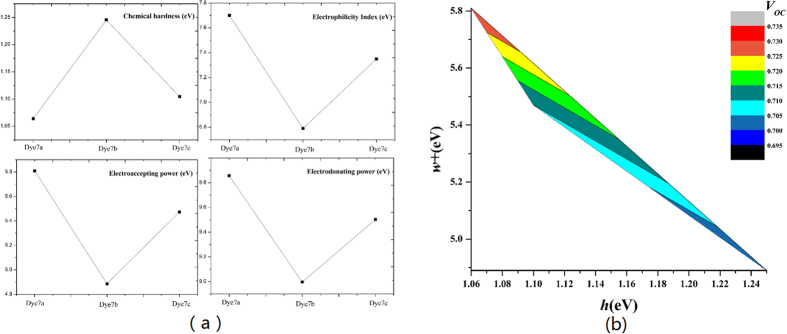
(**a**) Chemical hardness, electrophilicity index, electrodonating power and electroaccepting power calculated for dye7a, dye7b and dye7c. (**b**) Comparison graph *w* + vs. *h* and the colors map indicates the *V*_*OC*_ for the corresponding chemical parameters.

The chemical reactivity parameters using the ionic and neutral species, were shown in Table 5, and the graphics of chemical hardness (*h*), electronaccepting power (ω+), electrophilicity index (ω) and electrondonating power (ω−) were shown in Fig. 8.

The chemical hardness is an important factor that represents the resistance to intramolecular charge transfer^42^, and a lower chemical hardness is desired. Figure 8 (a) shows an order of dye7b(1.25 eV) > dye7c(1.10 eV) > dye7a(1.06 eV), indicating that 7a has the most advantage in charge transfer. Electronaccepting power represents the capability to accept electron, and 7a(5.81 eV) was significantly better than 7b(5.47 eV) and 7c(4.89 eV), which indicates a higher ability to attract the electrons from the donor fraction. Also electrophilicity shows results with a similar tendency to that of ω+, and thus 7a presents the highest energetic stability by acquiring electron from environment. As for electrondonating power, the lower value represents the higher capability of donating electrons, and the tendency is 7a(9.86 eV) > 7c(9.50 eV) > 7b(9.00 eV). The smallest electrondonating power for 7b may be attributed by planarization structure. 7a had obvious advantages in chemical reactivity parameters, and 7c performed better than 7b in *h*, ω and ω+. Furthermore, it can be seen in Fig. 8(b) that along with increasement of *h, V*_oc_ was decreased. When the value of ω+ was increased, the *V*_oc_ was also increased. Namely, the lower *h* and higher ω+ could lead to better *V*_oc_.

### Molecular design

#### Substitution of alkyl chains

Twisting geometry structure often affects the electron structure and spectra^43^, and for DSSCs, alkyl chains not only play a role of preventing the molecular aggregates, but also regulate the distortion degree. In order to analyze the effect of butyl on the dye molecule, we use the hydrogen atom to replace butyl (labeled as dye_h), named by 7a_h, 7b_h and 7c_h, to theoretically simulate the influence of alkyl chain on the dye properties.

Table 1 shows that after replaced by hydrogen atom, the |C1-C2-C3-C4| was decreased to be 0.61° from 19.82° for dye 7a_h and to 38.18° from 65.42° for dye 7c_h. For the dihedral angles formed between chromophore and triphenylamine units, |C5-C6-C7-C8| fell to 43.17° from 76.93° for 7a_h, and |C9-C10-C11-C12| was decreased to 9.87° from 39.78° in 7c_h; as for 7b_h, |C5-C6-C7-C8| and |C9-C10-C11-C12| were decreased to be 43.11° from 62.31° and to be 7.61° from 39.16°, respectively. It can be seen that the butyl orientation significantly regulated the geometric structure of original molecule, and this twisted both sides groups can affect the charge transfer between the donor and the acceptor.

**Figure 9:**
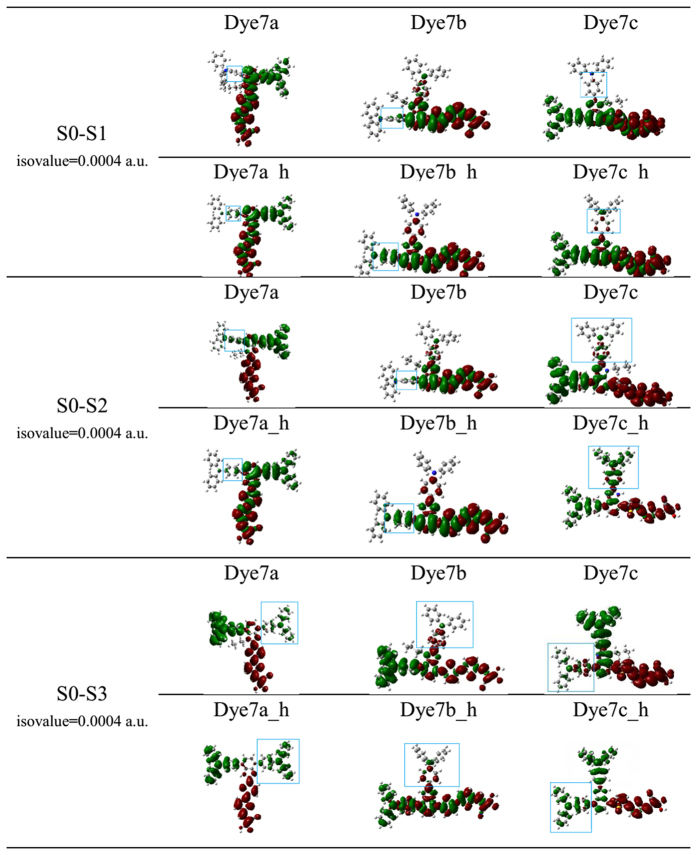
Electron density difference plots of electronic transition S0 → S1, S0 → S2 and S0 → S3 for all the dyes in Gas.

To directly show the intramolecular charge transfer more, we have simulated the charge density difference (CDD) in the excited states of S0-S1, S0-S2 and S0-S3, as shown in Fig. 9. Compared between original molecules and designed molecules, the distribution of electron and hole density were obviously decreased on that triphenylamine moiety towards alkyl chain, and there were more evidently blocking effect on the linear structure like 7b and 7c; while for dye 7a_h performed little density difference because of its unique T-shaped cross-conjunction nature. It can be concluded that the substitution of butyl can affect the charge-transfer properties.

**Figure 10:**
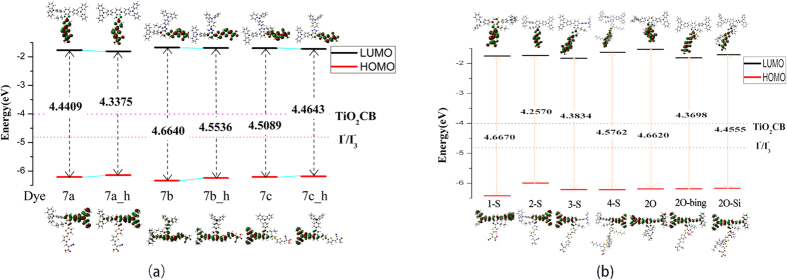
(**a**) Schematic energy levels of sensitizers, together with the valence band of TiO_2_ and I^^−^^/I_3_^^−^^ redox level in Gas. (**b**) Schematic energy levels for designed dyes in THF solvent.

**Figure 11:**
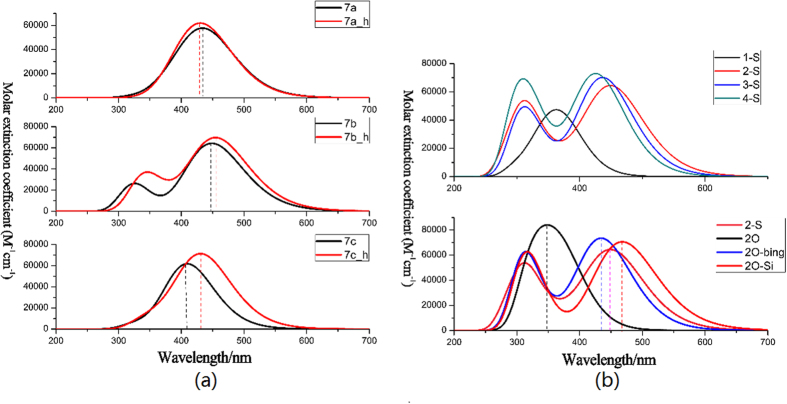
(**a**) Simulated absorption spectra obtained in Gas. (**b**) Simulated absorption spectra calculated for designed dyes in THF solvent.

Figure 10 shows the energy level of designed molecules. As shown in Fig. 10(a), it was seen that for 7a_h, 7b_h and 7c_h, the energy levels of LUMO were decreased compared with original molecules, and HOMO performed slightly upward. Absorption spectra were simulated, as shown in Fig. 11(a).

It seemed that the substitution of the butyl has little influence on absorption peak for T-type molecules, i.e., 439 nm for 7a and 438 nm for 7a_h, respectively. The obvious change of absorption was found for the substitution of planar structure on conjugated bridge, and the change was about 22 nm. Furthermore, a lower energy gap resulted generally in the change of absorption peak, which were supported by the simulated absorption spectra (see Fig. 11(a)).

#### Substitution of π-bridge

Dye7a, dye7b and dye7c possessed the same accepter, donor, π bridge and even chromophore. Although the T-shaped 7a assembly obtained the highest conversion efficiency (5.01%), the absorption spectra of 7a is blue-shifted compared with the planar linear arrangement 7b. It is well known a suitable π-conjugated linker could broaden absorption band and suppressed the intermolecular aggregation by the steric hindrance^44^. In the framework, the further design and optimization will be performed for dye 7a to adjust the linker conjugation by means of the insertion of an election-rich fusing ring thienothiophene (TT)^45^ or an 3,4-ethylenedioxythiophene (EDOT)^46^, which have been successfully coupled in other dye molecular and performed well.

**Figure 12:**
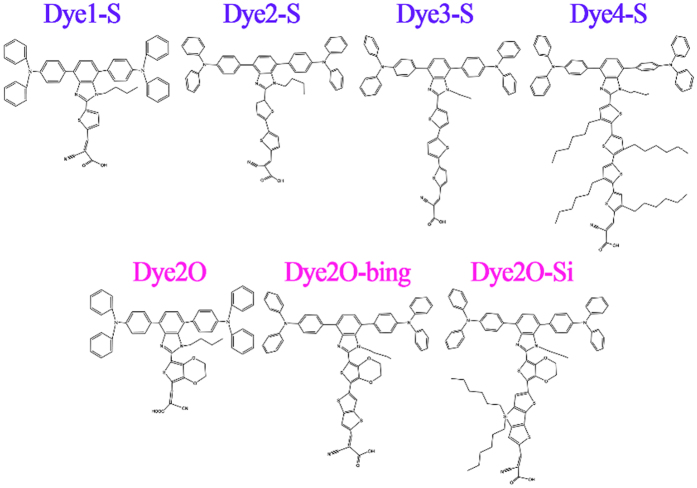
Names and chemical structures of the designed sensitizers.

Figure 12 shows the six designed molecules. Specifically, we considered the influence of the number of thiophene in bridge on performance of dye7a, and the designed dyes were named after dye1-S (containing one thiophene), dye3-S (containing three thiophenes) and dye4-S (containing four thiophenes), respectively. Another three designed molecules pointed to the substitute with 3,4-ethylenedioxy thiophene-category in bridge, which were classified into the dye2O, dye2O-bing and dye2O-Si, respectively. Molecular orbital energies and energy gap were shown in Fig. 10(b), the HOMO energy levels of the seven molecules are in this order: dye7a(dye2-S) > dye2O-Si > dye2O-bing > dye2O > dye4-S ≈ dye3-S > dye1-S, and the LUMO energy levels are in this order: dye3-S < dye2O-bing < dye1-S < dye7a < dye2O-Si < dye4-S < dye2O, indicating dye7a performed best in the HOMO levels; but dye3-S, dye2O-bing and dye1-S created the advantage in LUMO levels. Next, the energy gaps will be disused by category. It can be seen that the thiophene-category linker molecules were followed this order of dye1-S(4.6670 eV) > dye4-S(4.5762 eV) > dye3-S (4.3834 eV) > dye7a(4.2570 eV), and the dyes possessing 3,4-ethylenedioxy thiophene-category linker exhibited a trend of dye2O (4.6620 eV) > dye2O-Si (4.4555 eV) > dye2O-bing (4.3698 eV). Furthermore, from Figure S3 the lengths of π-conjugated linkers followed the tendency of dye4-S(16.85 Å) > dye3-S(13.07 Å) > dye7a(9.24 Å) for thiophene-category, and dye2O-Si(13.00 Å) > dye2O-bing(11.22 Å) for 3,4-ethylenedioxy thiophene-category, which corresponded well to the orders of energy gaps, respectively. However, the too short linker, such as dye1-S and dye2O, will significantly increase the values of energy gap.

Frontier molecular orbitals for all designed molecules were shown in Figure S4. It can be seen from Figure S4 that the main occupied molecular orbitals stays localization due to T-shape arrangement; for the HOMOs and HOMO-1s, they primarily located on two triphenylamine moieties, and LUMOs and LUMO+1 s chiefly located through cyanoacrylic acid and thiophene groups, except for dye1-S and dye2O. Noted that for dye1-S, the LUMO+1 spread on the triphenylamine moiety, and the LUMO+1 of dye2O is delocalized over the whole molecular, which both were not conducive to intramolecular charge transfer and probably led to the blue-shifted in spectra. In addition, the distribution of analysis of C+ and C− indicates that the two main excited states for dye1-S and dye2O were also different, and charge centroid centers were not orientated on their π-bridges (see Supporting materials in Figure S5).

The theoretical ultraviolet-visible absorption of the designed T-shaped dyes are shown in Fig. 11(b). It is observed that the absorption peaks for four dyes with thiophene-category linker exhibits the trend of dye7a > dye3-S > dye4-S > dye1-S, which is line with the tendency of energy gaps, namely the larger this energy gap is and this hypsochromic shift will be the more obvious^47^. Dye7a performed better than other dyes possessing thiophene-category linker in absorption spectrum. Compared the dye7a to the three dyes possessing 3,4-ethylenedioxythiophene-category linker, the absorption wavelength of dye2O-Si (468.02 nm) is larger than that of dye2-S. It also has largest absorption peak among the dyes with 3,4-ethylene dioxythiophene-category, and the molar extinction coefficient is also increased significantly. Some other parameters of dye7a and dye2O-Si were estimated to judge whether substitutions of T-shaped molecular through bridge could further improve the performance. key parameters were listed in Table 6. Calculated value of *LHE* exhibited an order: dye2O-bing(0.9842) > dye2O-Si(0.9819) > dye4-S(0.9836) > dye3-S(0.9810) > dye7a(0.9723) > dye2O(0.9256) > dye1-S(0.6761). Therefore, dye 2O-Si has outstanding ability of solar cell utility (*LHE* is increased from 0.9723 to 0.9819), and utility of sunlight can enhance the *J*_*SC*_. Meanwhile, the injection time (*τ*_inj_) decreased in this order of 2-S < 2O-Si < 4-S < 1-S < 2 O < 3-S < 2O-bing, and it is obvious that the *τ*_inj_ of 7a and 2O-Si has similar values which are faster than other designed dyes.

The vertical dipole moment for dye2O-Si is found to be 13.4956 D that is larger than dye7a, meaning that the *V*_oc_ can be improved by the introduction of 3,4-ethylenedioxythiophene.

In short, dye2-S(dye7a) had the optimal spectral response in thiophene-category linker dyes. As the increasing numbers of thiophene, *E*_gap_ was enlarged, and then the hypsochromic shift trend was more obvious. As for dye2O-Si with 3,4-ethylenedioxy thiophene-category linker, it showed a bathochromic shift of 16.71 nm compared with dye7a, and the molar extinction coefficient value were moderate to higher value (ranging from 64496 to 70634 *M*^−1^ *cm*^−1^). Furthermore, 2O-Si also possesses an ideal *τ*_inj_, the well results of *LHE*, vertical dipole moment and *λ*_e_ compared with dye7a. This configuration with the big linkers (like dye2O-Si) is also expected to suppress intermolecular aggregation by means of its steric hindrance^48^.

## Discussion

We perform a self-contained simulation on optic-electronic performance of the typical T-shaped and linear dyes to reveal the relationship between structure and performance. Ground structure, energy levels and gaps, the absorption spectra and the electronic properties were calculated with DFT and TD-DFT methods. Functional correction and solvent effect were considered and discussed. We also calculated the light harvesting efficiency (*LHE*), the driving force of electron injection (Δ*G*^*inject*^) and the electron injection time (*τ*_inj_), the excited state lifetime (τ), the vertical dipole moment (*μ*_normal_), chemical hardness (*h*), electronaccepting power (ω+), electrophilicity index (ω) and electrondonating power (ω−) as well as NBO analysis. Charge difference density coupled with index of spatial extent was used to study the charge transfer process, and the interaction model and strength between dyes and iodine were discussed. The following conclusions can be drawn from the calculated results: firstly, T-shaped arrangement of dye 7a performed excellent orbital localization for HOMO and LUMO, respectively. CDDs and index of spatial extent (S and D) methods demonstrated that the charge transfer for T-shaped dye 7a is better than the linear dye 7b. For the two types of dyes, absorption peaks of them make red-shifted along with the decreasing solution polarity. Secondly, calculations on the microscopic parameters relevant to *V*_oc_ and *J*_*SC*_ showed that there is little change for light harvesting efficiency (*LHE*), electron injection energy (Δ*G*^*inject*^) and electron regeneration energy(∆*G*^^reg^^), and the largest value of *J*_*SC*_ for dye 7a can be contributed to the fastest injection time (*τ*_inj_) and the longest excited lifetime for T-type dye 7a. Furthermore, the largest performance of *V*_oc_ is originated from the largest dipole moment (*μ*_normal_) for T-type 7a and its obvious chemical reactivity parameters (*h*, ω+, ω and ω−). We also found another way that lost electron and caused the low *V*_oc_. Discussion of three interaction models and NBO indicated that dye7a can inhibit the loss of electron. Finally, study of the substitution of alkyl chains showed that substitutions in donor unit not affected the absorption, and changes of spectra came from substitution in conjugated bridge. For designed molecules, the adjustment of π-bridge by increasing numbers of thiophene and 3,4-ethylenedioxy thiophene-category linker showed that the designed way by means of modification of 3,4-ethylenedioxythiophene-category is proved to be very effective for broadening absorption range, improving LHE, vertical dipole moment, etc. It is expected that among designed dyes, dye2O-Si possess better photoelectric properties by molecular regulation.

## Methods

All calculations were performed by using the Gaussian 09 program^49^. The ground-state geometric structures and natural bond orbital (NBO) were optimized by the CAM-B3LYP^20^ functional with 6–31G(d) basis set for C, H, O, N, S, I and Si atoms, and a LANL2DZ represents an effective core potential (ECP) for Ti atom (optimized ground-state geometric structures, see Supporting materials in Table S4). Frequency calculations are carried out for all the geometry optimizations to make sure no imaginary, that is, the geometry is in a global minimum. Their electronic excited states were calculated at the same level for molecules. In order to quantitatively describe the mechanism of ICT, three related parameters including S (overlap integral between C+ and C−), D (the distance of electron transfer [Å]) and Δq (the fraction of electron exchange (|e-|)) were calculated by code Multiwfn 3.3.9^50^. As for chemical reactivity parameters, they were calculated using the formulae reported by Parr and Pearson^51^ and Gázquez *et al*.^52^, from the ionic and neutral states energy values. The results are obtained by TD-DFT CAM-B3LYP/6-31G(d) in THF solvent.

The overall efficiency of photo-to-electron conversion in DSSCs is determined by the open–circuit photo voltage (*V*_oc_), fill factor (FF) and incident solar power on the cell^53^:


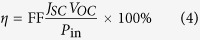


Where *J*_*SC*_ is the integral of short-circuit photocurrent density, which depends on the absorption coefficient of the dye and the interaction between the dye and the nanocrystalline TiO_2_ surface. It can be determined by using the following equation^54^,^55^:


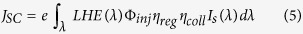


Where the parameter Φ_*inj*_ is the electron injection efficiency, and *η*_*coll*_ is the efficiency of electron collection. *LHE (λ*) is the light-harvesting efficiency at a maximum wavelength, which can be calculated by using the following expression^56^:


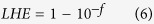


Where *f* is the oscillator strength at the maximum absorption (*λ*_max_) in the equation. Noticed that the larger *f* implies better harvesting of sunlight. At the same time, the quantum yield of electron Φ_*inj*_ can be related to the free-energy change during the electron-injection process. This free-energy change can be obtained using injection driving force (Δ*G*^*inject*^)^57^,^58^:


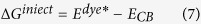


Where *E*^dye^^*^ is the oxidation potential energy of the dye in the excited state, and *E*_*CB*_ is the reduction potential of the conduction band of TiO_2_. The value of *E*_*CB*_ used in this work is −4.00 eV, which is widely used in some papers. And the *E*^dye^^*^ can be estimated by following equation^59^:


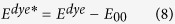


Where *E*^^*dye*^^ is the redox potential of the ground state of the dye, and *E*_00_ is the lowest vertical transition energy. And *η*_*reg*_ can be also measured by the driving force of regeneration (∆*G*^^reg^^) between the oxidized dye and electrolyte, and *E*^^*redox*^^ is the Fermi levels of electrolyte iodine/iodide, as can be calculated via the following expression


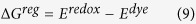


Furthermore, the Marcus electron transfer theory has proved that the total reorganization energy could also affect the kinetics of electron inject, which can be expressed as ref. 60:


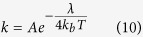


where *A* is a prefactor, *k*_*b*_ is the Boltzmann constant, T is the temperature, and *λ* is the total reorganization energy^60^,^61^ (the hole reorganization energy *λ*_*h*_ and the electron reorganization energy *λ*_*e*_), which can be expressed by the following formulas:


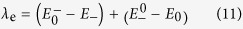



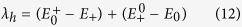


where 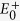 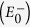 represents the energy of cation (anion) calculated from the optimized structure of the neutral molecule; *E*_+_ (*E*_−_) is the energy of cation (anion) calculated from the optimized cation (anion) structure; 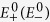 is the energy of neutral molecule calculated at the cationic (anionic) state; *E*_0_ is the energy of the neutral molecule at the ground state. The reorganization energy is the energy change which is caused by the change of the molecular configuration. Injection time of electron can be quantitative calculated from the Newns–Anderson model^34^,^35^, which is discussed in Results to describe the difference of the objected system.

## Additional Information

**How to cite this article**: Xu, B. *et al*. Photoactive layer based on T-shaped benzimidazole dyes used for solar cell: from photoelectric properties to molecular design. *Sci. Rep.* **7**, 45688; doi: 10.1038/srep45688 (2017).

**Publisher's note:** Springer Nature remains neutral with regard to jurisdictional claims in published maps and institutional affiliations.

## Supplementary Material

Supporting Information

## Figures and Tables

**Table 1:** Critical dihedral angles in both Gas and THF states.

Dyes	|C1-C2-C3-C4|		|C5-C6-C7-C8|		|C9-C10-C11-C12|	
	Gas	THF	Gas	THF	Gas	THF
7a	19.82	29.18	76.93	70.16	33.60	35.38
7a_h	0.61	2.26	43.17	42.27	32.64	34.17
7b	6.07	4.30	62.31	61.04	39.16	41.77
7b_h	7.20	3.68	43.11	41.85	7.61	5.14
7c	65.42	65.43	33.64	36.62	39.78	42.68
7c_h	38.18	37.50	32.61	35.28	9.87	8.25

**Table 2:** Oscillator strengths (*f*), Absorption peak *λ*_max_ (nm) and CI expansion coefficient (P)^a^.

Dye		Dominant Configuration	CI Expansion coefficient (P = 2|CI|^2^)	Absorption peak *λ*_max_(nm)	Strength *f*	Exp^b^ (nm)
**7a**	S1	H-2- > L	0.6031(73%)	451.31	1.5569	428
	S2	H- > L	0.5917(70%)	371.15	0.3023	
	S3	H-1- > L	0.6851(94%)	333.29	0.0009	
	S4	H- > L + 1	0.3847(30%)	320.05	0.7722	
	S5	H-4- > L	0.4042(33%)	307.19	0.4384	
	S6	H-3- > L	0.5635(64%)	293.00	0.0062	
**7b**	S1	H- > L	0.5155(53%)	473.44	1.6875	469
	S2	H-1- > L	0.4885(48%)	347.03	0.3747	
	S3	H-1- > L	0.3488(24%)	331.28	0.1471	
	S4	H-1- > L + 1	0.1966(8%)	314.71	0.1189	
	S5	H-1- > L	0.3165(20%)	302.98	0.7166	
	S6	H-4- > L	0.3671(27%)	293.58	0.2896	
**7c**	S1	H-2- > L	0.5863(69%)	423.97	1.6348	418
	S2	H- > L	0.4953(49%)	348.61	0.1890	
	S3	H-1- > L	0.6000(72%)	324.44	0.2038	
	S4	H- > L + 1	0.4533(41%)	322.92	0.7094	
	S5	H-1- > L + 1	0.4131(34%)	304.69	0.7867	
	S6	H-3- > L	0.3242(21%)	289.06	0.0222	

^a^The dominant configurations with larger configuration interaction (CI) expansion coefficients for the excited states. P = 2|CI|^2^ in parentheses represents the percentage contribution for the corresponding one-electron transition to each excited state. H stands for HOMO, and L stands for LUMO.

^b^Taken from ref. 12.

**Table 3:** Critical parameters (in eV) influencing *J*_*SC*_.

	Δ*G*^*inject*^	*E*^dye*^	*∆G*^*reg*^	*LHE*	*λ*_h_	*λ*_e_	τ(ns)	*E*_*LUMO*_	*τ*_inj_(fs)	*J*_*SC*_^a^
**7a**	−0.5604	3.4396	−2.2009	0.9723	0.23	0.48	2.64	−3.1929	3.96	9.42
**7b**	−0.2018	3.7982	−2.3626	0.9795	0.43	0.44	2.57	−3.0607	5.00	8.84
**7c**	−0.6781	3.3219	−2.2113	0.9768	0.21	0.48	2.26	−3.1175	4.89	6.92

^a^Taken from ref. 12, unit in (mA cm^−2^).

**Table 4:** Critical parameters influencing *V*_oc._

Dye	*μ*_normal_/D	Atomic charge on the atom interacting with I_2_ (in a.u.)			*V*_oc_^a^/V	*η*^a^(%)
		I1^*…*^I2	I3^*…*^I4	I5^*…*^I6		
**7a**	12.52	−0.00478	−0.05982	−0.17548	0.73	_5.01_
**7b**	11.15	−0.02644	−0.30596	−0.25136	0.70	_4.13_
**7c**	2.12	−0.02990	−0.27259	−0.23171	0.71	_3.45_

^a^Obtained from ref. 12.

**Table 5:** Chemical reactivity parameters for dye7a, dye7b and dye7c (in eV).

Molecule	I	A	*h*	ω	ω+	ω−
**Dye7a**	5.11	2.98	1.06	7.70	5.81	9.86
**Dye7b**	5.36	2.87	1.25	6.79	4.89	9.00
**Dye7c**	5.13	2.92	1.10	7.35	5.47	9.50

**Table 6:** Critical parameters influencing *V*_oc_ and *J*_sc_.

Dye	*λ*_max_(nm)	Δ*G*^*inject*^	*LHE*	*f*	*τ*_inj_(fs)	*μ*_normal_/D	*λ*_h_	*λ*_e_
**1-S**	339.90	−1.228	0.6761	0.4896	5.76	6.8481		
**2-S**^a^	451.31	−0.7542	0.9723	1.5569	3.96	12.5154	0.23	0.48
**3-S**	437.30	−0.6225	0.9810	1.7221	8.21	11.7661		
**4-S**	426.59	−0.7012	0.9836	1.7854	5.65	4.3240		
**2O**	325.38	−1.6174	0.9256	1.1284	6.69	11.6048		
**2O-bing**	435.23	−0.6632	0.9842	1.8009	10.34	11.7018		
**2O-Si**	468.02	−0.4821	0.9819	1.7432	4.11	13.4956	0.24	0.41

^a^Dye2-S is the original dye7a. ^b^Unit in eV.
