# Supplementary material for: Photoactive layer based on T-shaped benzimidazole dyes used for solar cell: from photoelectric properties to molecular design
Source: Sci Rep. 2017 Mar 28;7:45688. doi: 10.1038/srep45688 (PMC5368572; doi:10.1038/srep45688)
Supplement: Supporting Information [file srep45688-s1.pdf]

Supporting information

Photoactive layer based on T-shaped benzimidazole dyes used for solar cell: from photoelectric properties to molecular design

Beibei Xu[a], Yuanzuo Li[a,*], Peng Song[b,c], Fengcai Ma[c], Mengtao Sun[b,c,d,*]

[a] *College of Science, Northeast Forestry University, Harbin 150040, Heilongjiang, China;*

[b] *Beijing Key Laboratory for Magneto-Photoelectrical Composite and Interface Science, School of Mathematics and Physics, University of Science and Technology Beijing, Beijing 100083, China;*

[c] *Department of Physics, Liaoning University, Shenyang 110036, Liaoning, China;*

[d] *Beijing National Laboratory for Condensed Matter Physics, Beijing Key Laboratory for Nanomaterials and Nanodevices, Institute of Physics, Chinese Academy of Sciences, Beijing 100190, People's Republic of China;*

Content:

Figure S1. Electron density difference plots electronic transition for isolated dyes. S2
Figure S2. Calculated vertical dipole moment on TiO_2 surface. S3
Figure S3. The lengths of π spacers obtained from the optimized designed dyes. S4
Figure S4. The frontier molecular orbitals for designed dyes. S5
Figure S5. Computed difference in the centroids of hole and charge (C+/C-). S6
Table S1. Maximum absorption wavelength (λ_{max}) data using different methods. S7
Table S2. Transition electric dipole moments. S7
Table S3. The distances of I-X (X=I, N and S) for isolated dyes. S7
Table S4. Cartesian coordinates and total energies for 7a, 7b and 7c. S8

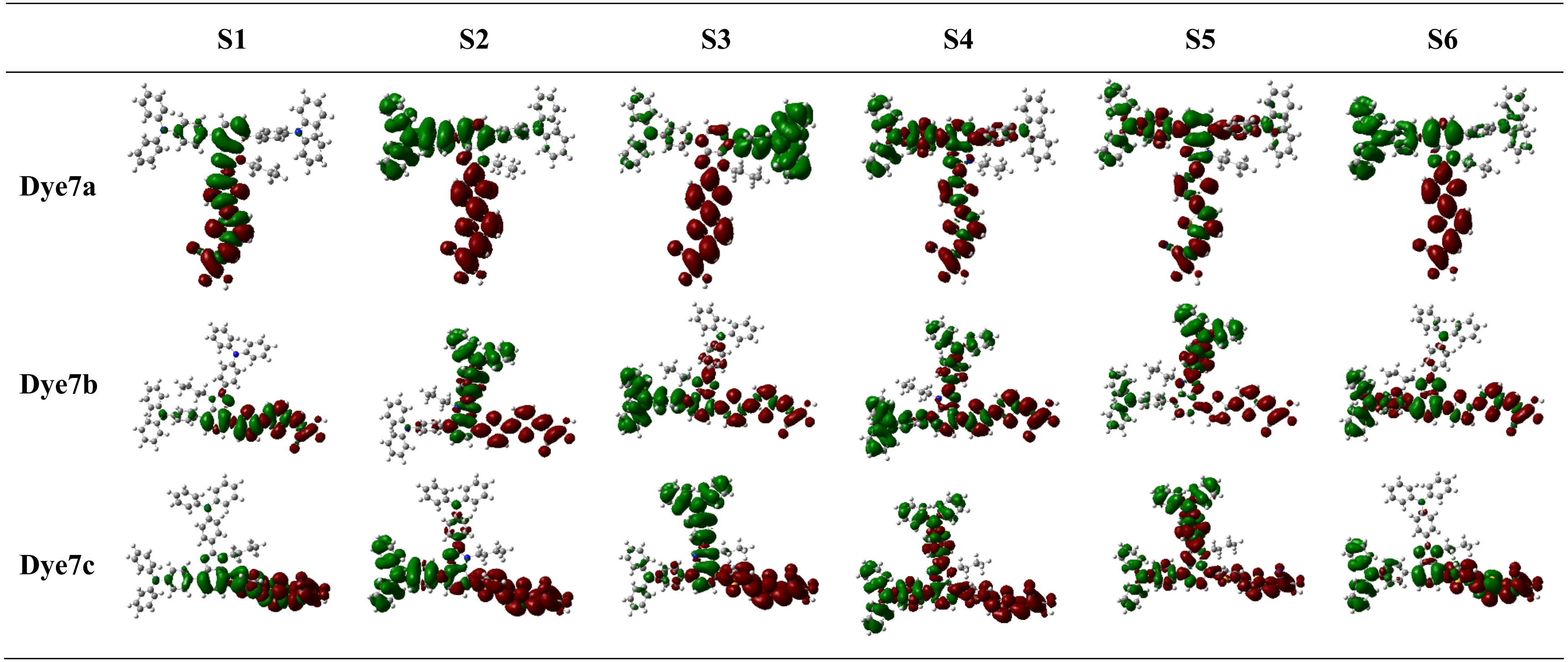

Figure S1. Electron density difference plots electronic transition for isolated dyes.

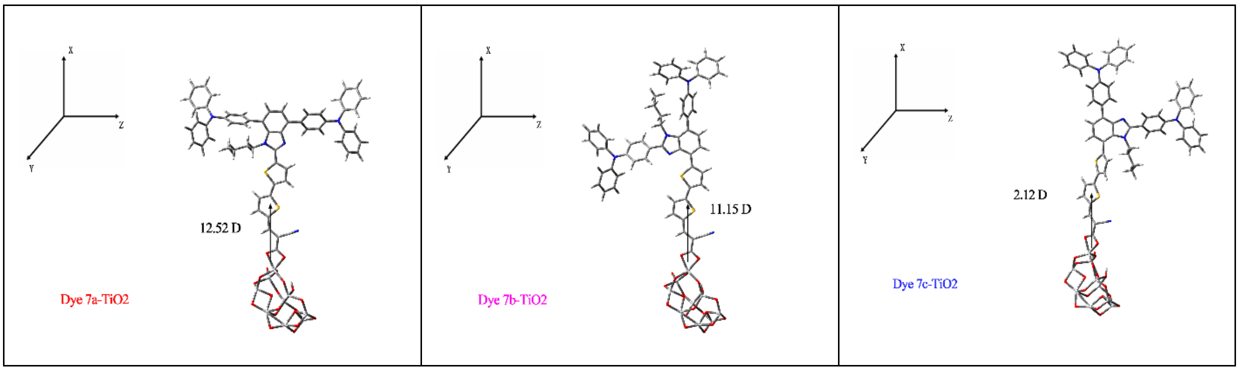

Figure S2. Calculated vertical dipole moment on TiO_2 surface.

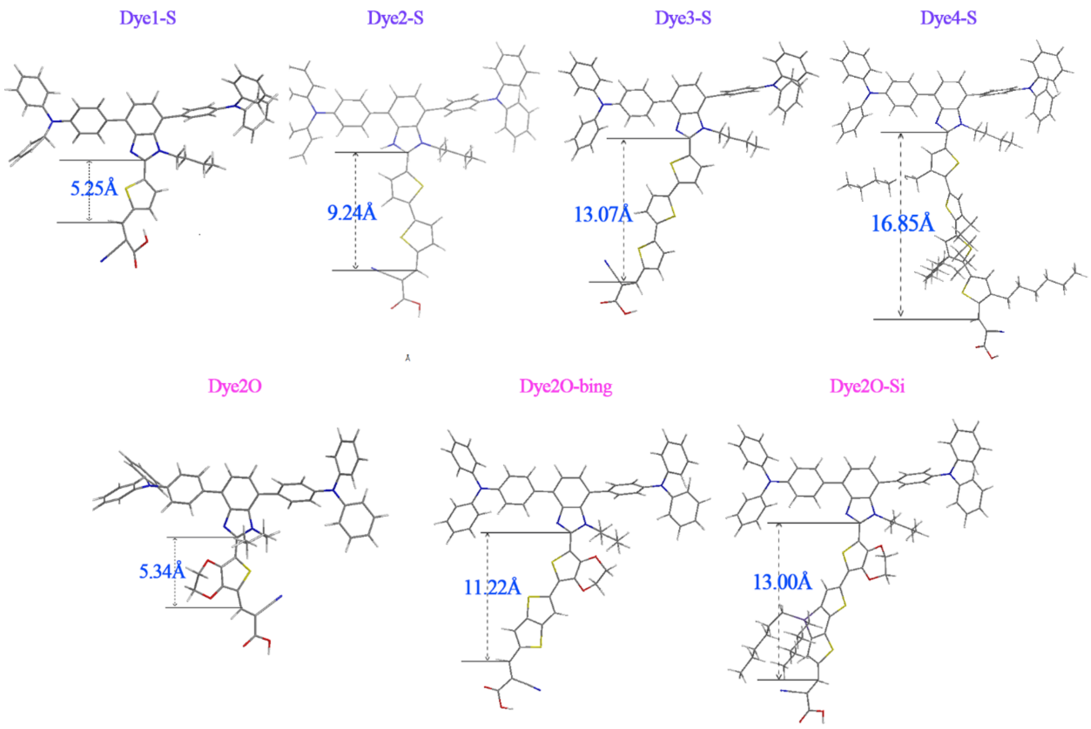

Figure S3. The lengths of π spacers obtained from the optimized designed dyes.

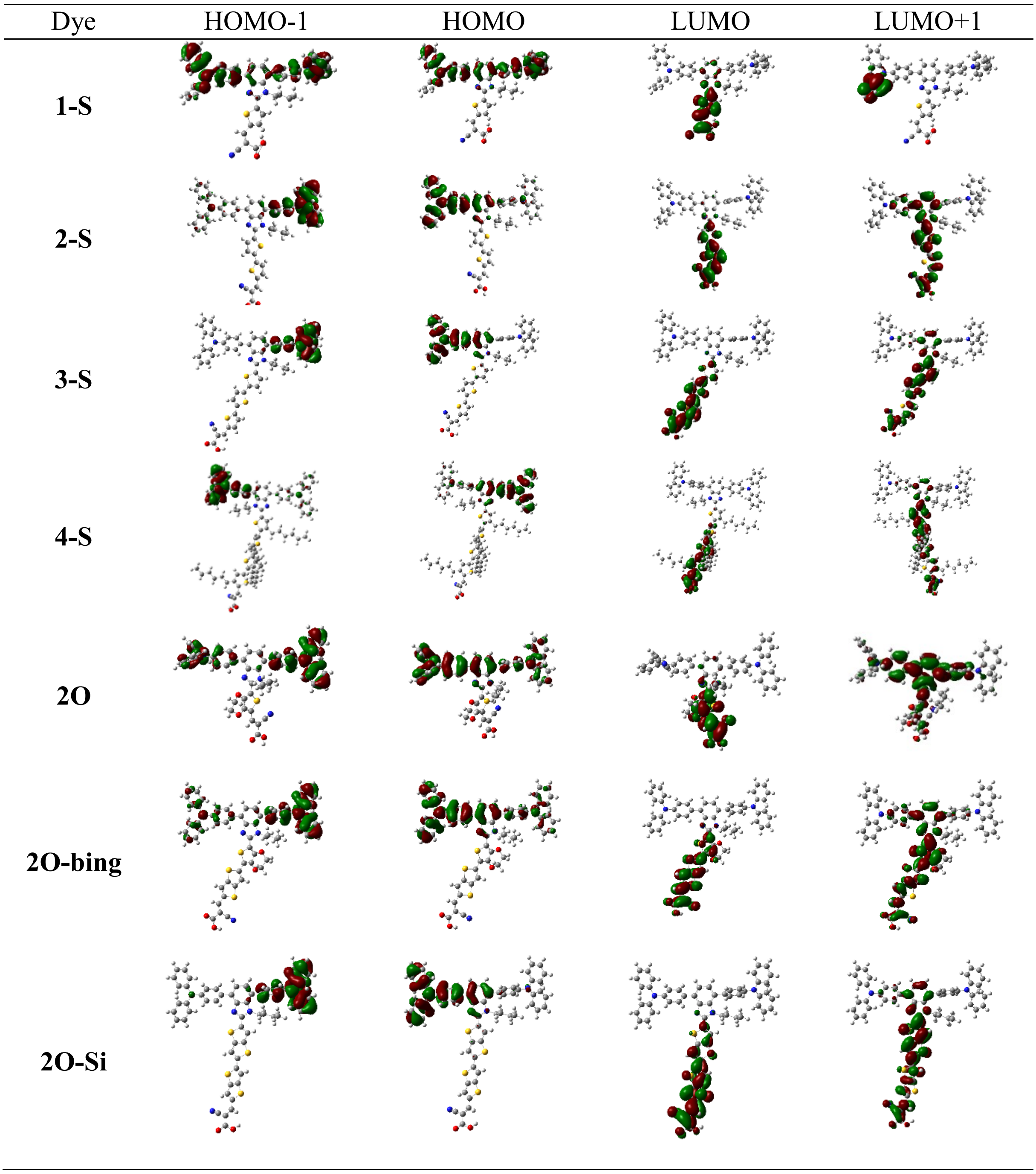

Figure S4. The frontier molecular orbitals for designed dyes.

Dye1-S	Dye2-S	Dye3-S	Dye4-S
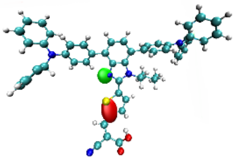<br>S0-S1<br>C_+: isovalue=0.0008 a.u.<br>C_- : isovalue=-0.00048 a.u.	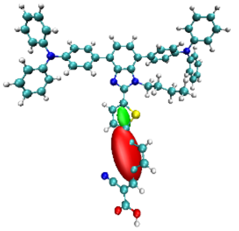<br>S0-S1<br>C_+: isovalue=0.000493 a.u.<br>C_- : isovalue=-0.000493 a.u.	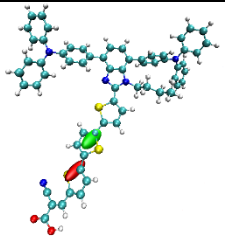<br>S0-S1<br>C_+: isovalue=0.00052 a.u.<br>C_- : isovalue=-0.00047 a.u.	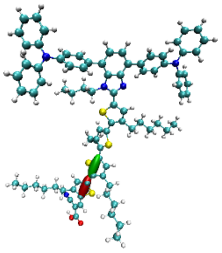<br>S0-S1<br>C_+: isovalue=0.0006 a.u.<br>C_- : isovalue=-0.00048 a.u.
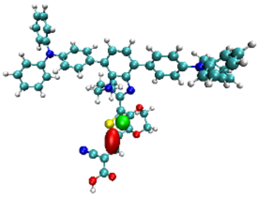<br>S0-S3<br>C_+: isovalue=0.001 a.u.<br>C_- :isovalue=-0.0008a.u.	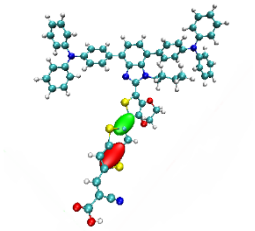<br>S0-S1<br>C_+: isovalue=0.00052 a.u.<br>C_- :isovalue=-0.00041a.u.	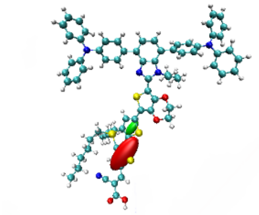<br>S0-S1<br>C_+: isovalue=0.000493 a.u.<br>C_- :isovalue=-0.000493 a.u.	
Dye2O	Dye2O-bing	Dye2O-Si	

Figure S5. Computed difference in the centroids of hole and charge (C+/C-).

Table S1. Maximum absorption wavelength (λ_{max}) data using different methods.

	B3LYP	CAM-B3LYP	ωB97xD	Exp[a]
Dye7a	519.01	451.31	439.55	428
Dye7b	515.27	473.44	461.01	469
Dye7c	475.53	423.97	404.83	418

Data [a] was taken from Ref[1]

Table S2. Transition electric dipole moments.

		X	Y	Z
7a	S1	-1.6494	-4.5178	-0.0229
	S2	1.9211	0.0452	-0.0245
	S3	0.0871	-0.0307	-0.0369
	S4	-2.4930	1.3851	0.0568
	S5	2.0791	-0.3125	0.1151
	S6	0.0833	0.2291	-0.0125
7b	S1	4.9722	1.2553	0.0544
	S2	1.1159	1.7423	-0.0108
	S3	1.2000	0.3957	0.0878
	S4	-0.0723	1.1073	0.0054
	S5	-1.3643	-2.2991	0.0044
	S6	1.6566	-0.2189	0.0825
7c	S1	4.7610	-0.3690	0.1188
	S2	0.6508	-1.3187	-0.0789
	S3	0.6036	1.3460	-0.0213
	S4	1.5259	-2.2827	0.0432
	S5	2.2113	1.7315	0.0558
	S6	-0.3717	0.1594	-0.2191

Table S3. The distances of I-X (X=I, N and S) for isolated dyes.

Dyes···I_2	CN···I1	I1···I2	$S_{thiphene1}$···I3	I3···I4	$S_{thiophene2}$···I5	I5···I6
**7a**	2.700	2.877	3.352	2.858	3.295	2.864
**7b**	2.670	2.882	3.305	2.864	3.160	2.886
**7c**	2.690	2.879	3.337	2.860	3.329	2.862

Table S4. Cartesian coordinates and total energies

Dye 7a(total energy= -3494.49877308 a.u)

C	0.33420100	0.29858400	-0.07668600
N	1.10280800	-0.77700500	-0.09632200
N	-1.02005400	-0.01286500	-0.06780100
C	0.25121300	-1.85207600	-0.07991000
C	-1.09996000	-1.40623400	-0.07235700
C	0.58381300	-3.23076500	-0.06329500
C	-2.18751600	-2.30628400	-0.06615600
C	-0.50377700	-4.10702600	-0.01801000
C	-1.83229100	-3.65881500	-0.02158100
H	-0.31997400	-5.17542900	0.03824900
H	-2.63261000	-4.39349300	-0.00381700
C	-2.14155800	0.91586400	0.08581900
C	-2.52444900	1.16769600	1.55054700
H	-2.99308000	0.50343400	-0.45543400
H	-1.87920600	1.84989600	-0.41790400
C	-3.71599000	2.12457400	1.68008800
H	-2.76977900	0.20651600	2.01912000
H	-1.65971200	1.57227600	2.09283100
C	-4.11202900	2.38693000	3.13651900
H	-3.47586600	3.07781700	1.18764600
H	-4.57459800	1.70468200	1.13727800
H	-4.96474800	3.07207000	3.19947100
H	-4.39397300	1.45602000	3.64266400
H	-3.28270700	2.83260400	3.69885100
C	-3.63562300	-1.94871800	-0.12429800
C	-4.47896700	-2.18029000	0.97327500
C	-4.22299500	-1.45436200	-1.30119300
C	-5.84316600	-1.90541200	0.91323800
H	-4.05421800	-2.56774000	1.89565200
C	-5.58787100	-1.19300900	-1.37842600
H	-3.60338000	-1.30103900	-2.18140900
C	-6.42170900	-1.40996800	-0.26739600
H	-6.46800200	-2.07756500	1.78352900
H	-6.01779800	-0.83105600	-2.30662700
N	-7.81126200	-1.14440800	-0.33942300
C	-8.73953700	-2.00329600	0.31629700
C	-8.29385500	-0.02996500	-1.08175000
C	-9.81077500	-1.46375900	1.04525800
C	-8.60088900	-3.39803700	0.23837000
C	-9.41457700	-0.16407700	-1.91662100
C	-7.66361300	1.22089700	-0.98500700
C	-10.72577000	-2.30523300	1.67545400
H	-9.92030800	-0.38589800	1.11178500
C	-9.51015500	-4.23141900	0.88739100
H	-7.77973500	-3.82171700	-0.33124400
C	-9.89441200	0.93219400	-2.63090300

H	-9.90440200	-1.12907900	-1.99954300
C	-8.13942800	2.30708600	-1.71707300
H	-6.80318700	1.33550500	-0.33284800
C	-10.57953000	-3.69224200	1.60569100
H	-11.54995700	-1.87146800	2.23564300
H	-9.38846200	-5.30914300	0.81723700
C	-9.25888000	2.17238100	-2.54091900
H	-10.76323800	0.81012300	-3.27233500
H	-7.63954500	3.26830100	-1.62966700
H	-11.29047400	-4.34487700	2.10419500
H	-9.63156400	3.02282500	-3.10453200
C	1.97512300	-3.73242200	-0.09021800
C	2.28111800	-4.95328200	-0.72024100
C	3.03615500	-3.04206000	0.52484600
C	3.57279600	-5.46504200	-0.73538100
H	1.49979800	-5.49505600	-1.24575900
C	4.32777200	-3.55603200	0.52966600
H	2.83954500	-2.09692400	1.01587000
C	4.62006500	-4.77623700	-0.10154800
H	3.77970100	-6.39801500	-1.24975900
H	5.12158000	-3.01213700	1.03180300
N	5.93954700	-5.29707200	-0.10592200
C	6.15521200	-6.69194600	0.06501400
C	7.05010700	-4.42655100	-0.28677400
C	7.10468000	-7.36877300	-0.71776000
C	5.42313500	-7.41494100	1.02107000
C	8.20938700	-4.58047000	0.49038200
C	7.00469900	-3.40212500	-1.24606700
C	7.31917700	-8.73411600	-0.53912300
H	7.67029300	-6.81878300	-1.46316200
C	5.63198700	-8.78352700	1.17961400
H	4.69318500	-6.89776600	1.63571300
C	9.29954800	-3.73302000	0.30200200
H	8.24829400	-5.36574700	1.23863800
C	8.09310900	-2.54826600	-1.41445100
H	6.11414900	-3.28126900	-1.85456200
C	6.58286600	-9.45215400	0.40562900
H	8.05816000	-9.24095100	-1.15439000
H	5.05628900	-9.32621600	1.92502700
C	9.24858300	-2.70963700	-0.64683800
H	10.18873900	-3.86638100	0.91276600
H	8.04065500	-1.76058400	-2.16160300
H	6.74815100	-10.51770400	0.53717400
H	10.09741600	-2.04617900	-0.78558600
C	0.95009000	1.61854300	-0.10255200
C	2.29859500	1.80915900	-0.35334300
S	0.17954600	3.15665400	0.23160800
C	2.70670800	3.15516000	-0.29082600
H	2.95140200	0.97444700	-0.57393500
C	1.68321500	4.03591700	0.01458600

H	3.72713200	3.47709600	-0.47057200
C	1.72797400	5.46839200	0.15010200
C	0.68378700	6.34256300	0.43466100
S	3.23510400	6.33546900	-0.04463300
C	1.08800600	7.68336300	0.49359600
H	-0.33761800	6.01403100	0.59191000
C	2.44658700	7.88040300	0.25775700
H	0.41579100	8.50935800	0.70240100
C	3.07291200	9.15981700	0.26676300
C	4.37806800	9.52071200	0.05488000
H	2.39175500	9.97993200	0.47770800
C	5.40735500	8.57611000	-0.23544700
C	4.81580600	10.93519000	0.11242300
N	6.22632800	7.78231100	-0.47113000
O	5.95440900	11.31301400	-0.06632500
O	3.79690700	11.79385100	0.39119400
H	4.20247900	12.68002900	0.40247500

Dye 7b(total energy= -3494.50524186 a.u)

C	-0.11630700	0.55352900	0.08023700
N	0.78582500	-0.40893700	0.01936300
N	-1.41558700	0.05959000	0.09543900
C	0.08367000	-1.58594800	0.01147600
C	-1.31282800	-1.33308500	0.04071300
C	0.61566000	-2.89559200	0.01044900
C	-2.25586400	-2.38290600	0.02981100
C	-0.33137000	-3.93001500	0.05007100
C	-1.70437500	-3.67424600	0.05582700
H	-0.00299400	-4.96368700	0.06512400
H	-2.39040700	-4.51640000	0.04935500
C	-2.64404200	0.85475300	0.14834200
C	-3.30370900	0.88577200	1.53335300
H	-3.34696100	0.45237700	-0.58369600
H	-2.38496700	1.86497300	-0.17626700
C	-4.54107900	1.79214800	1.55652200
H	-3.58720300	-0.13389000	1.81871900
H	-2.57557900	1.22665200	2.28181800
C	-5.24511600	1.80288700	2.91723400
H	-4.24995900	2.81696800	1.28459900
H	-5.24534800	1.45919600	0.78119900
H	-6.11989000	2.46266800	2.90831400
H	-5.58637000	0.79785600	3.19226400
H	-4.57191500	2.15295600	3.70914400
C	0.26856600	1.97476700	0.07714200
C	-0.33830000	2.96624900	0.86646800

C	1.36286300	2.35638700	-0.72113100
C	0.11160500	4.28182600	0.84575100
H	-1.13943800	2.70827500	1.55069500
C	1.80800000	3.66976700	-0.75868600
H	1.85231300	1.60174600	-1.32770100
C	1.18659700	4.66180100	0.02358000
H	-0.36103800	5.02074700	1.48390900
H	2.63886200	3.93813800	-1.40242500
N	1.63511600	5.99999900	-0.00975400
C	0.71086300	7.07569300	0.12949900
C	3.02012800	6.29402800	-0.17664500
C	1.00481000	8.15659100	0.97465700
C	-0.49709400	7.07573500	-0.58540900
C	3.42668900	7.29459200	-1.07228300
C	3.99170300	5.59776000	0.55873500
C	0.10740800	9.21612900	1.09666600
H	1.93728700	8.16010400	1.53024300
C	-1.39646900	8.13092400	-0.44342600
H	-0.72278300	6.24811100	-1.25061300
C	4.77953600	7.59425700	-1.22238800
H	2.67818700	7.83264400	-1.64543000
C	5.34350400	5.89197700	0.38879100
H	3.68187100	4.82919500	1.26002800
C	-1.09942000	9.20825000	0.39421900
H	0.34950500	10.04599700	1.75531200
H	-2.32721000	8.11699700	-1.00452100
C	5.74576400	6.89310800	-0.49791900
H	5.07873100	8.37197900	-1.92003600
H	6.08417200	5.34440300	0.96562200
H	-1.79915100	10.03275400	0.49658000
H	6.79970100	7.12479700	-0.62206700
C	-3.73823200	-2.25457200	-0.03987500
C	-4.54933200	-2.77902500	0.98041600
C	-4.38372700	-1.71266300	-1.16429900
C	-5.93837400	-2.73944700	0.89979100
H	-4.08010700	-3.20767400	1.86204700
C	-5.77178300	-1.68377200	-1.26357200
H	-3.78756600	-1.35118600	-1.99850000
C	-6.57444200	-2.19400400	-0.22850700
H	-6.53849900	-3.13521700	1.71264800
H	-6.24124200	-1.28176800	-2.15546200
N	-7.98673400	-2.17028200	-0.32561500
C	-8.75875700	-3.26453500	0.15960800
C	-8.64602200	-1.06755300	-0.93880400
C	-9.93122200	-3.03182500	0.89533700
C	-8.36521900	-4.58730800	-0.09722800
C	-9.68678000	-1.28166500	-1.85590200
C	-8.27088300	0.24929400	-0.62906900
C	-10.69498800	-4.10264500	1.35616100
H	-10.23800100	-2.01091300	1.10002600

C	-9.12566900	-5.65186700	0.38338100
H	-7.46481500	-4.77365800	-0.67407000
C	-10.33872100	-0.19918200	-2.44388400
H	-9.97895800	-2.29761500	-2.10195200
C	-8.91619700	1.32541600	-1.23545100
H	-7.47758700	0.42086800	0.09206000
C	-10.29600700	-5.41778300	1.10843000
H	-11.60042100	-3.90544600	1.92407800
H	-8.80739900	-6.67005500	0.17549300
C	-9.95565000	1.10954000	-2.14283700
H	-11.14254900	-0.38271500	-3.15194300
H	-8.61395300	2.33871100	-0.98384900
H	-10.89000400	-6.24990300	1.47530400
H	-10.46176100	1.95055100	-2.60801900
C	2.04854900	-3.16898100	-0.02081200
C	2.66043600	-4.41299100	-0.08397300
S	3.26521700	-1.90389700	0.02724400
C	4.06644500	-4.36537400	-0.09005800
H	2.10622900	-5.34283800	-0.13011400
C	4.57115100	-3.07668800	-0.03281300
H	4.69290500	-5.25029800	-0.13720800
C	5.94621100	-2.65595900	-0.02336400
C	6.45070900	-1.35859500	0.03334200
S	7.24592800	-3.82755800	-0.08654100
C	7.84980600	-1.30968800	0.02586100
H	5.81530600	-0.48126600	0.07903100
C	8.46711500	-2.55852900	-0.03616400
H	8.42329500	-0.38892500	0.06452400
C	9.87666300	-2.74119600	-0.05423300
C	10.63703400	-3.88282500	-0.11270400
H	10.43880300	-1.81188200	-0.01450000
C	10.07111100	-5.19110600	-0.17024000
C	12.11540600	-3.83378200	-0.11992600
N	9.58413500	-6.24826200	-0.21641000
O	12.84167000	-4.80507100	-0.16981800
O	12.60629800	-2.56342800	-0.06381200
H	13.57543200	-2.66479400	-0.07471700

Dye 7c(total energy= -3494.50176867 a.u)

C	0.69028600	0.81001600	-0.11720400
N	1.54086000	-0.19489100	-0.14380000
N	-0.63538600	0.38985000	-0.19593900
C	0.77833200	-1.33794800	-0.22236400
C	-0.60318900	-1.00177700	-0.27750600
C	1.22673700	-2.68036600	-0.23336100
C	-1.60211700	-1.99845300	-0.38623500
C	0.22029900	-3.65285500	-0.28882400
C	-1.13469400	-3.32285000	-0.36365100

H	0.49454600	-4.70258400	-0.26016500
H	-1.86547600	-4.12306300	-0.43571100
C	-1.81541300	1.25619900	-0.20524900
C	-2.57337400	1.28867700	1.12839000
H	-2.48465500	0.92133700	-0.99939600
H	-1.47446200	2.25779000	-0.47580800
C	-3.79262800	2.21857800	1.07315100
H	-2.89553200	0.27296300	1.38635100
H	-1.89347200	1.60809800	1.92993400
C	-4.56788600	2.26238700	2.39377700
H	-3.46748200	3.23409000	0.80494000
H	-4.46206900	1.88779500	0.26733100
H	-5.43350900	2.93002900	2.32316200
H	-4.93539600	1.26666700	2.66871600
H	-3.93496600	2.62130500	3.21454100
C	1.14944800	2.20915900	-0.06630700
C	0.55331500	3.21761400	0.70920800
C	2.30702700	2.54386300	-0.79288700
C	1.07413600	4.50683900	0.74145500
H	-0.29918600	2.99188700	1.34107100
C	2.82348800	3.83144200	-0.77843700
H	2.79014500	1.77255300	-1.38334500
C	2.21243500	4.84187800	-0.01201500
H	0.60609500	5.25932100	1.36710800
H	3.70335700	4.06499400	-1.36830600
N	2.73151000	6.15544100	0.00601300
C	1.85529700	7.27483900	0.10596800
C	4.13660700	6.37906500	-0.07813000
C	2.15271300	8.33137700	0.98030100
C	0.69125600	7.34180700	-0.67553800
C	4.64374600	7.38342700	-0.91679300
C	5.02967000	5.60777900	0.68194000
C	1.30279000	9.43288900	1.06467700
H	3.05090400	8.28315700	1.58786700
C	-0.16203100	8.43883400	-0.57134700
H	0.46251100	6.53250800	-1.36183600
C	6.01666800	7.61311200	-0.98589600
H	3.95741100	7.97947400	-1.50992100
C	6.40233800	5.83185200	0.59162800
H	4.64295900	4.83522800	1.33904300
C	0.13916300	9.49200900	0.29499700
H	1.54721600	10.24306900	1.74660400
H	-1.05918100	8.47612500	-1.18371800
C	6.90433200	6.83716800	-0.23763300
H	6.39346600	8.39502000	-1.63997600
H	7.08072400	5.22513000	1.18559000
H	-0.52416900	10.34906000	0.36810000
H	7.97430500	7.01395000	-0.29947600
C	2.65554800	-3.06038500	-0.18747500
C	3.10332700	-4.23395200	-0.82299600

C	3.61465900	-2.29618900	0.50384500
C	4.43362900	-4.63181500	-0.77154300
H	2.40508400	-4.82759900	-1.40603700
C	4.94315100	-2.69813700	0.57635500
H	3.30863200	-1.38075100	0.99457300
C	5.37731700	-3.87214800	-0.06067900
H	4.75136000	-5.52933700	-1.29246800
H	5.65605400	-2.09971000	1.13462600
N	6.73603000	-4.27559700	0.00380600
C	7.06564200	-5.65004300	0.15758100
C	7.77213900	-3.30579700	-0.09322000
C	8.12154800	-6.21724200	-0.57441700
C	6.34181600	-6.46166400	1.04646500
C	8.89616300	-3.37664100	0.74560700
C	7.68783000	-2.26396000	-1.03098100
C	8.44689700	-7.56250000	-0.41195200
H	8.68225400	-5.59814600	-1.26740200
C	6.66312300	-7.81013900	1.18824100
H	5.53014400	-6.02841000	1.62214500
C	9.91412400	-2.43091600	0.63844600
H	8.96505100	-4.17531200	1.47732700
C	8.70229400	-1.31263700	-1.11811500
H	6.82477900	-2.20553900	-1.68635400
C	7.71896700	-8.36934700	0.46516600
H	9.26698700	-7.98432500	-0.98730800
H	6.09200600	-8.42277300	1.88110000
C	9.82361800	-1.39113000	-0.28937100
H	10.77682900	-2.50152900	1.29593800
H	8.61983400	-0.51314400	-1.84987700
H	7.97122600	-9.41925600	0.58384200
H	10.61601400	-0.65190800	-0.36521300
C	-3.04972500	-1.77391800	-0.54718700
C	-3.74111100	-1.14112500	-1.55901400
S	-4.17527300	-2.47448800	0.60066700
C	-5.14987600	-1.20436500	-1.42182800
H	-3.24362400	-0.68725500	-2.40910700
C	-5.56690200	-1.90038100	-0.30279900
H	-5.84027500	-0.77894300	-2.14300300
C	-6.91158600	-2.16673200	0.14581000
C	-7.33098600	-3.04816800	1.13524700
S	-8.27094700	-1.32636300	-0.56402500
C	-8.72250900	-3.05592600	1.31181100
H	-6.64702800	-3.67464800	1.69698500
C	-9.40902400	-2.18512000	0.46939300
H	-9.23729200	-3.68425000	2.03154400
C	-10.82566600	-2.03329500	0.47404200
C	-11.64466300	-1.23360200	-0.27947600
H	-11.33123400	-2.66142600	1.20270600
C	-11.15358600	-0.34429300	-1.28162100
C	-13.11588500	-1.23635900	-0.10256000

N	-10.72374200	0.37288500	-2.09234300
O	-13.88857700	-0.55144200	-0.73900000
O	-13.53124400	-2.09979600	0.86427300
H	-14.50181300	-2.01374500	0.88735200

1. Bodedla, G. B., Thomas, K. R. J., Fan, M. S. & Ho, K. C. Benzimidazole-Branched Isomeric Dyes: Effect of Molecular Constitution on Photophysical, Electrochemical, and Photovoltaic Properties. *J. Org. Chem.* **81,** 640-653 (2016).
